# Frequent *ZNF217* mutations lead to transcriptional deregulation of interferon signal transduction via altered chromatin accessibility in B cell lymphoma

**DOI:** 10.1038/s41375-023-02013-9

**Published:** 2023-08-30

**Authors:** Franziska Briest, Daniel Noerenberg, Cornelius Hennch, Kenichi Yoshida, Raphael Hablesreiter, Jose Nimo, Daniel Sasca, Marieluise Kirchner, Larry Mansouri, Yoshikage Inoue, Laura Wiegand, Annette M. Staiger, Beatrice Casadei, Penelope Korkolopoulou, January Weiner, Armando Lopez-Guillermo, Arne Warth, Tamás Schneider, Ákos Nagy, Wolfram Klapper, Michael Hummel, George Kanellis, Ioannis Anagnostopoulos, Philipp Mertins, Lars Bullinger, Richard Rosenquist, Theodoros P. Vassilakopoulos, German Ott, Seishi Ogawa, Frederik Damm

**Affiliations:** 1 Department of Hematology, Oncology and Cancer Immunology, Campus Virchow, Charité – Universitätsmedizin Berlin, corporate member of Freie Universität Berlin and Humboldt-Universität zu Berlin, Berlin, Germany; 2 Department of Pathology and Tumor Biology, Graduate School of Medicine, Kyoto University, Kyoto, Japan; 3 Cancer Genome Project Wellcome Trust Sanger Institute, Hinxton, United Kingdom; 4 Department of Hematology, Oncology, and Pulmonary Medicine, University Medical Center, Johannes Gutenberg-University, Mainz, Germany; 5 Core Unit Proteomics, Berlin Institute of Health at Charité - Universitätsmedizin Berlin and Max-Delbrück-Center for Molecular Medicine, Berlin, Germany; 6 Department of Molecular Medicine and Surgery, Karolinska Institutet, Stockholm, Sweden; 7 Department of Clinical Pathology, Robert-Bosch-Krankenhaus, Stuttgart, Germany; 8 Dr Margarete Fischer-Bosch Institute of Clinical Pharmacology Stuttgart, and University of Tuebingen, Stuttgart, Germany; 9 IRCCS Azienda Ospedaliero-Universitaria di Bologna, Istituto di Ematologia “Seràgnoli”, Bologna, Italy; 10 First Department of Pathology, National and Kapodistrian University of Athens, Laikon General Hospital, Athens, Greece; 11 Core Unit Bioinformatics Berlin, Berlin Institute of Health at Charité - Universitätsmedizin Berlin, Berlin, Germany; 12 Hematology Department, Hospital Clinic, University of Barcelona, Barcelona, Spain; 13 Institute of Pathology, University Hospital Heidelberg, Heidelberg, Germany; 14 National Institute of Oncology, Budapest, Hungary; 15 HCEMM-SE Molecular Oncohematology Research Group, Department of Pathology and Experimental Cancer Research, Semmelweis University, Budapest, Hungary; 16 Department of Pathology, Hematopathology Section and Lymph Node Registry, Universitätsklinikum Schleswig-Holstein, Kiel, Germany; 17 Department of Pathology, Charité – Universitätsmedizin Berlin, corporate member of Freie Universität Berlin and Humboldt-Universität zu Berlin, Berlin, Germany; 18 German Cancer Consortium (DKTK) and German Cancer Research Center (DKFZ), Heidelberg, Germany; 19 Department of Hematopathology, Evangelismos General Hospital, Athens, Greece; 20 Institute of Pathology, University of Würzburg and Comprehensive Cancer Center (CCC) Mainfranken, Würzburg, Germany; 21 Clinical Genetics, Karolinska University Hospital, Stockholm, Sweden; 22 Department of Hematology and Bone Marrow Transplantation, National and Kapodistrian University of Athens, Laikon General Hospital, Athens, Greece; 23 Institute for the Advanced Study of Human Biology (WPI-ASHBi), Kyoto University, Kyoto, Japan; 24 Department of Medicine, Centre for Haematology and Regenerative Medicine, Karolinska Institutet, Stockholm, Sweden

**Keywords:** B-cell lymphoma, Cancer genetics, Oncogenes

## Abstract

Recent exome-wide studies discovered frequent somatic mutations in the epigenetic modifier *ZNF217* in primary mediastinal B cell lymphoma (PMBCL) and related disorders. As functional consequences of *ZNF217* alterations remain unknown, we comprehensively evaluated their impact in PMBCL. Targeted sequencing identified genetic lesions affecting *ZNF217* in 33% of 157 PMBCL patients. Subsequent gene expression profiling (*n* = 120) revealed changes in cytokine and interferon signal transduction in *ZNF217*-aberrant PMBCL cases. In vitro, knockout of *ZNF217* led to changes in chromatin accessibility interfering with binding motifs for crucial lymphoma-associated transcription factors. This led to disturbed expression of interferon-responsive and inflammation-associated genes, altered cell behavior, and aberrant differentiation. Mass spectrometry demonstrates that ZNF217 acts within a histone modifier complex containing LSD1, CoREST and HDAC and interferes with H3K4 methylation and H3K27 acetylation. Concluding, our data suggest non-catalytic activity of ZNF217, which directs histone modifier complex function and controls B cell differentiation-associated patterns of chromatin structure.

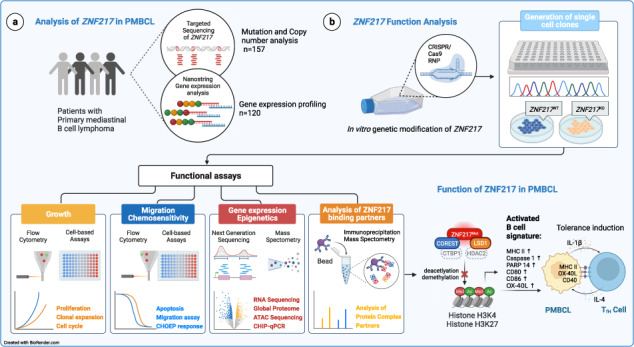

## Introduction

Lymphoid malignancies are frequently affected by somatic mutations in epigenetic modifiers regulating histone methylation, histone acetylation, and DNA methylation [1–3]. In various B cell lymphomas, such as diffuse large B cell lymphoma (DLBCL) or follicular lymphoma (FL), somatic mutations in KMT2D (*MLL2), EZH2, CREBBP*, and *TET2* are frequent events, affecting germinal center (GC) formation and affinity maturation [1, 4–6]. Our increasing knowledge on their function in regulation of differentiation and antigen expression [7–11], led to a better understanding of lymphomagenesis and new treatment options such as EZH2 inhibitors [12].

A large proportion of B cell lymphomas arise from GC B cells and often share dependence on the same transcriptional regulators (for example, BCL6). Transcriptional repression of gene promoters and enhancers involved in terminal differentiation, chemotaxis, and proliferation checkpoints is a central mechanism of transcriptional regulation. In addition to histone-modifying enzymes (KM2D, CREBBP, EP300), histone demethylases, especially KDM1A (LSD1), are required for GC formation and play a critical role in BCL6-driven lymphomagenesis [13]. LSD1 acts together with histone deacetylases (HDACs) and CoREST in transcriptional corepressor complexes. HDACs are involved in the development of various hematopoietic lineages and conditional knockout of HDAC3 in early progenitor B cells resulted in impaired B cell maturation and a defect in VDJ recombination in mouse models [14].

We and others recently reported recurrent mutations in the CoREST-binding protein ZNF217 in tumor samples from patients with primary mediastinal B cell lymphoma (PMBCL), gray zone lymphoma and Hodgkin lymphoma (HL) with the highest prevalence in PMBCL (~30%) [7, 15–20]. *ZNF217*, located on chromosome 20q13.2, has also been found among the 20 most frequently amplified genes in metastatic cancer [21].

To better understand ZNF217 involvement in lymphomagenesis, we performed a comprehensive genetic analysis in a large patient cohort of 157 primary PMBCL specimens coupled with functional analysis in two GC-derived lymphoma cell lines to unravel its function in the transcriptional deregulation of B cell lymphoma.

## Material and methods

### Patient samples

This study included 157 previously untreated PMBCL patients from collaborating centers in 5 countries. Additional 83 samples with available RNA and known *ZNF217* mutation status were studied [20, 22] (Supplementary Table 1).

In general, we included patients above the age of 18, if their clinical presentation (dominated by a mediastinal mass) and a reviewed histology report including estimation of tumor content (G.O., I.A., or T.V.) were consistent with the diagnosis of PMBCL according to the WHO [23]. Tumor purity was calculated using the *ALL-FIT* algorithm [24]. For FFPE tumor tissue, DNA was extracted using the Maxwell DNA-FFPE Kit (Promega, Madison, WI, USA). For fresh frozen tumor tissue (FF), genomic DNA was extracted using the Maxwell 16 Tissue DNA Purification Kit. RNA was isolated using the Maxwell® RSC RNA FFPE Kit.

The study was conducted in accordance with the Declaration of Helsinki. The protocol was approved by the ethics review committee of the Charité - Universitätsmedizin Berlin, and of every participating center. All patients gave written informed consent.

### Gene panel design for targeted sequencing

Based on published data and our ongoing genome/exome-wide sequencing studies (manuscript in preparation [20]), a designated panel of 106 genes targeting all coding regions was selected to generate a custom cRNA bait library (SureSelect, Agilent Technologies), which was used for targeted gene capture (Supplementary Table S2). In addition, a set of baits targeting common SNPs was designed to allow for copy-number calling. To improve the performance of the panel we added custom baits targeting regions that were poorly covered by conventional design and to genomic regions of high interest. Further information is supplied in Supplementary Table S2.

### Targeted sequencing

Library preparation for targeted sequencing was performed with the SureSelect custom capture bait kit (Agilent Technologies, Santa Clara, California, USA). To ensure high quality, only samples that had a coverage of 100x in ≥80% of the exonic regions were included. Variant calling was performed using Genomon2 pipelines (https://genomon.readthedocs.io/ja/latest/), as previously reported [25]. Specifics of the bioinformatic pipeline and detailed filtering criteria for detection of FFPE-derived artifacts and exclusion of germline variants are outlined in the Supplemental methods part. To overcome the limitations associated with the lack of germline DNA, we developed rigorous filtering criteria by comparison of tumor-only targeted sequencing data with paired tumor-germline whole-exome sequencing data in 57 PMBCL cases that were investigated with both techniques. Cross-validation rates WES/TS for SNVs and SCNAs were: 97% and 93% [20]. This trained algorithm was thereafter applied to all tumor-only cases presented in the present study. Two samples did not have any mutation in the 106 genes included in the sequencing panel. For more details refer to supplementary material and methods.

### Analysis of copy-number aberrations

SCNAs were evaluated on the basis of sequencing data using CNACS (https://github.com/papaemmelab/toil_cnacs), which also allowed for detection of copy-number neutral loss of heterozygosity likely caused by uniparental disomy (UPD). In case of multiple copy-number changes, the dominating SCNA was called for a given arm. All identified SCNAs were curated by visual inspection.

### Expression profiling with nCounter technology

The NanoString nCounter Flex system was used to run a customized version of the PanCancer Pathways Panel and 30 additional transcripts of interest (Supplementary Table S3). Genes were tested for differential expression in response to each selected covariate. For each gene, a single linear regression was fitted using all selected covariates to predict expression and false discovery rate (FDR) was estimated according to the Benjamini-Hochberg procedure. For further specifications refer to the supplementary material and methods section.

### Cell culture and treatments

The aggressive B-NHL cell lines Karpas1106P (RRID:CVCL_1821), Farage (RRID:CVCL_3302), U2940 (RRID:CVCL_1897), MedB-1 (RRID:CVCL_A649), RC-K8 (RRID:CVCL_1883) and the HL cell line L-428 (RRID: CVCL_1361) were purchased from Leibniz-Institute DSMZ-German Collection of Microorganisms and Cell Cultures, Braunschweig, Germany. Cells were cultured in IMDM + L-glutamine +phenol red (Thermo Fisher Scientific, Waltham, MA, USA), and 10–20% FBS (Sigma–Aldrich, St. Louis, MO, USA) and 1% penicillin/streptomycin. Cells were handled according to guidelines for the use of cell lines in biomedical research.

The components of the R-CHO(E)P treatments (doxorubicin, vincristine, cyclophosphamide, etoposide, prednisone, rituximab) were obtained pre-dissolved in saline from the Charité dispensary and mixed at a clinical ratio (Table S4). Proteasome inhibition was carried out by use of bortezomib (Velcade, Charité dispensary).

### Genetic modification of cell lines

The single guide RNAs (sgRNA) were designed using the CRISPRscan Platform (https://www.crisprscan.org/; accessed on 18.05.2018) and 3 different sgRNAs (Supplementary Table S5) with the best CRISPRscan scores were utilized [26]. PCR for sgRNA template amplification was performed with KAPA HiFi HotStart ReadyMix PCR Kit (KAPA Biosystems, Hoffmann-La Roche, Basel, Switzerland) and cleaned up with DNA Clean & Concentrator kit (Zymo Research Europe GmbH, Freiburg, Germany). In vitro transcription was performed with NEB HiScribe™ T7 High Yield RNA Synthesis Kit according to the manufacturer’s recommendations. The concentration of the resulting sgRNA products was adjusted to 1 μg/μL with RNase-free water. SgRNA was pre-complexed with resuspended Cas9 (1 μg/μL) with different ratios (1:3, 1:5, and 1:7). Cell lines were transfected with the Neon Transfection system (Thermo Fisher Scientific, Waltham, MA, USA; Supplementary methods) and clonal selection was performed by single cell dilution starting 24 h after transfection [27].

### WST-1 proliferation assay

5 × 10^3^ viable cells per clone were synchronized by serum starvation over 6 h, and 5 × 10^3^ were seeded in 5 replicates. For untreated proliferation assay, media was supplemented with additional 1% FBS every other day. For all treatment assays no additional FBS was added. For CD40 ligation, Ultra-LEAF™ Purified anti-human CD40 Antibody and Ultra-LEAF™ Purified Mouse IgG1, κ Isotype Control Antibody (Biolegend, San Diego, USA) were used at 1 µg/ml. 10 ng/ml of human recombinant IL-4 and IL-21 (R&D Systems, Inc., Minneapolis, USA) were added to the medium. For cell viability estimation after chemotherapy, 1.5 × 10^4^ viable cells were analyzed after 48 h of treatment with a serial dilution of a 100x R-CHOEP. Viable cell count was measured with WST-1 reagent (Sigma–Aldrich, St. Louis, MO, USA) as previously published in a spectrophotometer (Infinite 200 Pro M Plex, Tecan, Männedorf, Switzerland) [28]. Data was analyzed with Prism v9.1.0 (Graphpad Software, Inc., San Diego, CA, USA).

### Migration assay

1 × 10^5^ living cells were seeded into 5 µm pored Corning Transwell cell culture inserts with a chemotactic FBS gradient. After 24 h, cell suspensions in the lower chambers were analyzed on a Guava EasyCyte HT device (EMD Millipore Corporation, Hayward; CA, USA) according to the Manufacturer’s instructions.

### Clonal expansion assay

Cell lines were transfected with Cas9-RNP and cell bulk was cultured over an observation period of 5 to 11 weeks. PCR was performed to amplify a 531 bp regions of interest (ROI) within the *ZNF217* gene with the following M13-tagged primers:

fwd: 5′ tgtaaaacgacggccagtTCGCTTTTGATGTTGAGATCC 3′ and

rev: 5′ caggaaacagctatgaccTTTCTCCAAGCTCCTTCTCG 3′.

Indels were determined by Sanger sequencing and longitudinal analysis was carried out with the ICE online analysis tool [29].

### Determination of viability, cytotoxicity and apoptosis

Cells were cultured seeded in 1 µg/ml anti-human CD40 and 10 ng/ml IL-4 and IL-21 for 48 h. Medium was removed and 1.5 × 10^5^ cells were seeded into 96-well plates in replicates with anti-CD40 and IL-4/IL-21 or controls. ApoTox-Glo Triplex Assay (Promega, Walldorf, Germany) was applied for analysis according to the manufacturer’s recommendation.

### Flow cytometry

To determine cell viability, cell pellets were resolved in PBS containing 1% BSA and 5 µM diamidino-2-phenylindole (DAPI; BioLegend, San Diego, Ca, USA). Cells were analyzed by flow cytometry (BD FACSCanto II, BD Biosciences, Franklin Lakes, NJ, USA).

For apoptosis determination assay, 3 × 10^5^ cells were diluted in ice-cold 1x Annexin-V binding buffer and stained with anti-Annexin V-FITC (1:30; BD Pharmingen, BD Biosciences, Franklin Lakes, NJ, USA) and 5 µM DAPI. For determination of MHC class II presentation, 2 × 10^5^ cells were stained in PBS/1% BSA with the following antibodies (1:50): FITC Mouse Anti-Human HLA-DR, DP, DQ (RRID:AB_393926) and FITC Mouse IgG2a, κ Isotype Control, (RRID:AB_479604, both BD Biosciences, Franklin Lakes, NJ, USA) for 15 min., washed, and counterstained with DAPI. Cells were analyzed on FACSCanto II.

For cell cycle and mitotic index flow cytometry 3 × 10^5^ cells were analyzed as previously published [28].

### Fluorescence in-situ-hybridization (FISH)

FISH was performed with the CytoCell ZNF217 Amplification FISH Probe from Oxford Gene Technology (Begbroke, UK) according to the manufacturer’s recommendation with minor modifications in sample preparation and pre-denaturation.

### Western blot

Western blot was carried out using a standard wet blot protocol (for details refer to supplementary methods). The following antibodies were used for immunodetection: anti-GAPDH (RRID:AB_11174761), anti-beta-Actin (RRID:AB_2714189), anti-ZNF217 (RRID:AB_11127311), anti-CoREST (RRID:AB_2798514). Secondary antibodies were obtained from Dako (Agilent, Santa Clara, USA).

### Cloning and mutagenesis

*ZNF217* cDNA was generated by in vitro transcription and extension PCR and inserted into a pUC19 cloning vector for amplification. Restriction sites for further subcloning were added by PCR and fragment was transferred into a pcDNA3-EGFP mammalian expression vector after excision of eGFP. N-terminal flag, linker and stop site were induced by site-directed mutagenesis by use of Q5 Site-Directed Mutagenesis Kit (NEB, Germany). For detailed information refer to supplementary methods.

### RNA-sequencing

RNA-sequencing was carried out as previously published on a NextSeq500 platform (Illumina) in PE76 mode [27]. Differentially expressed genes were identified using the DESeq2 R package. Detailed information of RNA-seq and downstream analysis can be found in the supplementary methods section.

### ATAC-sequencing

1 × 10^5^ cells with >90% overall viability were used for lysis and transposase reaction. ATAC-sequencing was carried out by use of a previously published Omni-ATAC protocol [30] with minor modifications. PCR was performed with 5 cycles of pre-amplification using a common Ad1 primer and specific Ad2 primers for single-indexing based on Illumina Nextera i7 adapter barcodes (Supplementary Table S6). Number of additional cycles (*n* = 3) was determined by real-time qPCR. The final PCR product was cleaned up with AMPure XP magnetic beads (Beckman Colter, Brea, CA, USA). Sequencing was carried out on NextSeq500 High Output NGS platform (Illumina) in PE76 mode (see Supplementary Methods).

### Co-immunoprecipitation

Cells were harvested and lysed. Lysates were incubated under continuous rotation with prewashed Anti-FLAG® M2 Magnetic Beads (Sigma–Aldrich) at 4 °C overnight. To immobilize protein-bound beads, tubes were placed into a magnetic separation rack. Beads were washed twice with freshly prepared 1 ml TBS + 0.1% NP40 and twice with 1 ml TBS. Further details in supplementary methods.

### Mass spectrometry and interactome analyses

For global and phospho-proteome analysis, cell pellets were lysed in SDC lysis buffer and treated with Benzonase®. Protein digestion, TMT labeling, measurements and data analyses as described [31]. Phosphopeptide enrichment was performed using Fe(III)-IMAC cartridges and the AssayMAP Bravo Platform (Agilent Technologies). For the ZNF217 interactome pulldown samples were processed and analyzed as described [32] (see Supplementary Methods).

### Chromatin-immunoprecipitation followed by quantitative PCR (CHIP-qPCR)

CHIP was carried out by use of the iDeal ChIP-seq kit for Histones (Diagenode, Liège, Belgium) followed by standard qPCR. Please refer to the supplementary methods section and Supplementary Table S7 for further details.

### Statistical analyses

Mutation associations in primary material were analyzed with SPSS Statistics v26.0.0.0 and Prism 9.1.0. In vitro data was analyzed using Prism 9. The exact sample size (*n*) for each experimental group/condition is given in the figure legend. In general, parametric tests were chosen for datasets with assumption of normality. Normality was assumed after Shapiro–Wilk and Kolmogorov–Smirnov test. All differences were considered to be significant with alpha=0.05. Sample numbers were kept comparable to decrease heterogeneity of variances. If variances could not be assumed equal, Welch’s ANOVA or Geisser–Greenhouse correction was applied. Multiple testing correction was applied if applicable. Two-tailed tests were applied. All tests and applied correction are indicated in the figure legends. Growth curves were fitted with an exponential (Malthusian) growth model using least squares regression without further weighting. Extra sum-of-squares F test was used to compare non-linear regression data of cell proliferation.

## Results

### *ZNF217* is frequently altered in PMBCL and perturbation is associated with deregulation of genes involved in cytokine and interferon signaling

**Fig. 1:**
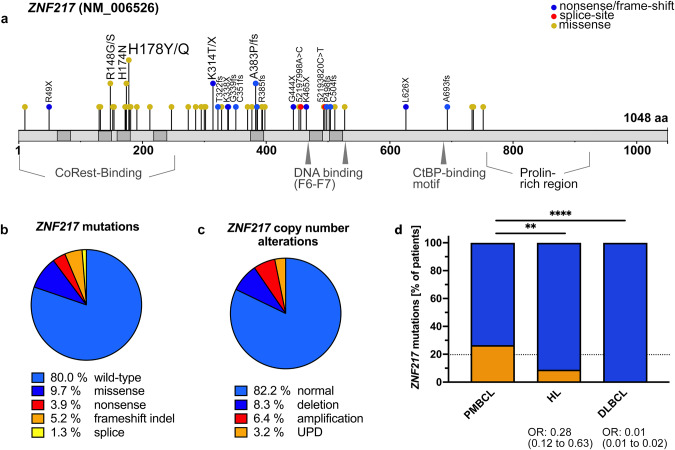
*ZNF217* aberrations in PMBCL patients. **a** Mutation diagram (lolliplot) for *ZNF217*. 31/157 PMBCL samples (19.7%) harbored *ZNF217* SNVs or indels. Hot-spot mutations, truncating mutations (blue) and splice mutations (red) were labeled with specification of the alteration, SNVs are visualized in yellow. Domain data was obtained from Nunez et al. [55] and https://www.uniprot.org/. **b** Distribution of mutation types and **c** CNA of *n* = 157 PMBCL patients. In case of >1 alteration, the most deleterious type was attributed to the patient. **d** Comparison of *ZNF217* mutation prevalence in PMBCL, HL and DLBCL. Patient-level data of *ZNF217* was extracted from our data set and previous studies in PMBCL [7, 15], HL [17–19], and DLBCL [56–58]. Merged data were compared by two-sided fisher’s exact test. 95% CI of OR was calculated by Baptista-Pike method. Individual prevalence of the mutated gene in the present study is indicated by the dashed line.

**Fig. 2:**
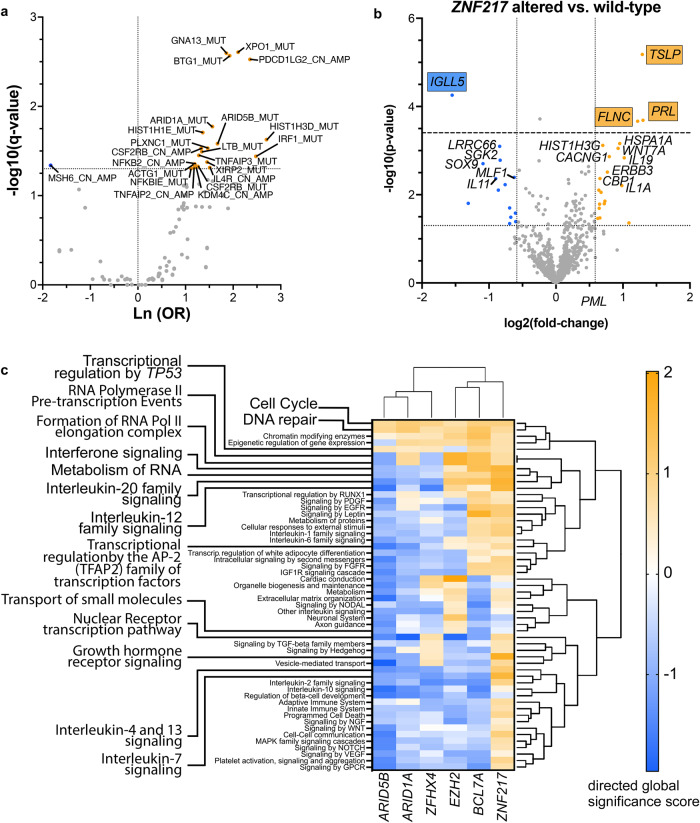
Gene expression in *ZNF217* altered PMBCL. **a** Volcano plot showing systematic evaluation of pairwise associations between mutated *ZNF217* and mutations or copy-number alterations in other genes of the targeted panel. 2 × 2 contingency tables were generated for each pair followed by Fisher’s exact tests and adjustment for multiple testing using Benjamini-Hochberg correction. y-axis displays the *q*-value of each pair as -log10(*q*) (dotted line indicates *q* < 0.05). x-axis represents the strength of the correlation expressed as log odds where blue dots indicate mutually exclusive and orange dots co-mutated genomic lesions. **b** RNA was isolated from *n* = 120 PMBCL patients and analyzed with the human nCounter PanCancer Pathways panel probe set (Nanostring). Volcano plot shows differentially higher (orange) and lower (blue) expressed transcripts with their log_2_-fold change on the x-axis. Y-axis shows -log10(*p*) (dotted line indicates *p* < 0.05; dashed line indicates FDR < 0.05). **c** Differential expression at gene set level. Global directed gene expression heatmap on gene set level, bearing either ≥1 copy-number changes and/or ≥1 SNV in either *ARID5B*, *ARID1A*, *ZFHX4*, *EZH2*, *BCL7A*, or *ZNF217* versus respective wild types demonstrated substantial differences in the global perturbation patterns induced by *ZNF217* alterations. Gene expression was analyzed by use of an extended nCounter PanCancer probe panel (Nanostring technologies). Directed global significance score was calculated using the Nanostring nSolver 4.0.70 software as the square root of the mean signed squared *t*-statistic for the genes in a gene set, with t-statistics coming from the linear regression underlying the differential expression analysis. Pathways with the highest and smallest differences in pathway scores between *ZNF217* and *ARID1A* were highlighted.

Targeted sequencing of 157 PMCL samples was performed with median and mean coverages of 592x and 684x, respectively (219x − 2573x, Supplementary Tables S1 and S2). A total of 57 ZNF217 variants in 31 patients with an average VAF of 23.7% (range 3.3–75.3%) were identified. Among these 57 variants, 41 were non-synonymous missense, 14 were frameshift/stopgain and two were splice sites mutations (Fig. 1). With mean PolyPhen-2 HVAR and HDIV scores of 0.70 and 0.78, and a mean SIFT score of 0.10, 61%, 71%, and 54% of *ZNF217* missense mutations were predicted as damaging, respectively. Missense mutations predominantly and spatially clustered in hot-spot regions located in the C2H2 domains. Putative inactivating alterations (e.g., frameshift, splice site, or nonsense mutations) were spread over the entire coding region, showing a mutation pattern highly reminiscent of a tumor suppressor gene (Fig. 1a). Comparison of the overall prevalence of *ZNF217* mutations found in this and two other PMBCL studies with data reported in HL and DLBCL, indicate an enrichment of *ZNF217* mutations in PMBCL (Fig. 1d). In addition to recurrent mutations, copy-number alterations (CNAs) were detected in 28/157 (18%) of the patients (Fig. 1, Supplementary Fig. S1). Of those 13 (8.3%) were deletions, 10 (6.4%) were amplifications and 5 were identified as uniparental disomy (UPD; 3.2%), leading to an overall frequency of *ZNF217* alterations (mutation and/or CNA) of 52/157 (33%, Fig. S1a). To identify interactions of *ZNF217* genetic aberrations with other mutations, we performed co-occurrence and exclusivity analysis using Fisher exact tests (Fig. 2a). A remarkable number of positive correlations with other genomic aberrations was observed, most notably mutations in *GNA13, XPO1, BTG1* and amplifications in *PDCD1LG2* (*PD-L2*).

Next, we compared the gene expression pattern of 120 PMBCL samples using the nCounter targeted transcriptional profiling platform from NanoString. Patients with *ZNF217* alterations (*n* = 53; mutation and/or CNA) showed a significantly higher mRNA expression of the hemopoietic cytokine thymic stromal lymphopoietin (*TSLP*), prolactin (*PRL*), and the actin-binding filamin c (*FLNC*). *IGLL5*, which has been described as frequently mutated in other lymphomas [7, 15], was identified as significantly downregulated in *ZNF217* altered specimens (Fig. 2b, Supplementary Table S2). Mono- or bi-allelic loss of* ZNF217* was rare and did not affect gene expression significantly. Alterations in *ZNF217* resulted in increased gene expression activity affecting the majority of pathways covered by the NanoString panel. Especially genes associated with interleukin and interferon signal transduction were identified as upregulated showing a distinct gene expression pattern as compared with other altered chromatin remodelers (Fig. 2c, Supplementary Fig. S1b). In addition, a CD40 activation-like signature was identified and validated in in vitro experiments (Supplementary Fig. S1c)

### Disruption of *ZNF217* in vitro reveals ZNF217 as a global transcriptional regulator of interferon-responsive signaling associated with B cell activation

In non-hematologic cancers, DNA binding of ZNF217 to specific cis-regulatory regions correlates negatively with gene expression, suggesting its function as a transcriptional repressor through interaction with epigenetic regulators such as CoREST, CtBP, LSD1, and EZH2 [33]. In order to gain functional information about ZNF217 in lymphoma, we analyzed the *ZNF217* status in cell lines of GC origin. All available PMBCL cell lines harbored at least one altered *ZNF217* allele, ranging from a heterozygous S513P mutation with predicted damaging consequence in Farage cells, a copy-number deletion in Karpas1106P to (bi-)allelic loss in MedB-1 and U2940 by frameshift alterations (Supplementary Fig. S2). To analyze the role of *ZNF217* in GC-derived aggressive lymphoma, we therefore selected two lymphoma cells lines without *ZNF217* deletions/mutations, L-428 and RC-K8 [34, 35]. We generated a large number of different *ZNF217* knockout single cell clones by use of a CRISPR/Cas9-based genome editing protocol (Supplementary Fig. S2).

**Fig. 3:**
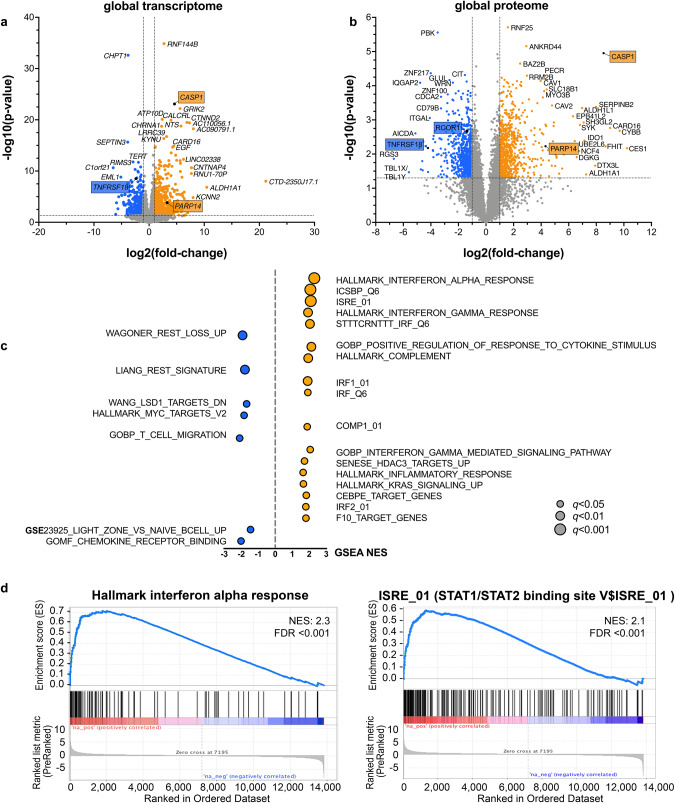
Gene expression after knockout of *ZNF217* in aggressive lymphoma cell lines. **a** RC-K8 cells (ZNF217^KO^: *n* = 7; ZNF217^WT^: *n* = 4) were bulk sequenced by RNA-Sequencing for transcriptome analysis. Genetic modification of *ZNF217* resulted in large changes in gene expression triggering the significantly altered expression of 1022 genes (log_2_-fold change <-0.5/ > 0.5; *q* < 0.05). Genes chosen for further evaluation by CHIP-*q*PCR are marked. **b** Additional analysis of the global proteome of ZNF217^KO^ (*n* = 5) versus ZNF217^WT^ cells (*n* = 4) verified the data obtained from transcriptomic analysis with high consistency. Proteins subjected to further analyses are marked. **c** Pre-ranked gene set enrichment analysis (GSEA) was performed including the following gene sets collections from https://www.gsea-msigdb.org: MSigDB: (H) Hallmark, (C5) GO, (C3) TFT, (C2) CP: REACTOME, custom DB (defined Supplementary Table S8). Genes having at least one occurrence of transcription factor binding motifs of STAT1, IRF8, C/EBP-epsilon, IRF1 or IRF2, respectively, were significantly enriched [59]. Enrichment data for each enriched gene set can be found in Supplementary Table S8. Graphs show normalized enrichment scores (NES) and false discovery rate (FDR) *q*-values of GSEA analysis. **d** Enrichment plots for two of the top-ranked gene sets Hallmark Interferon-alpha response and ISRE_01 are shown in detail. ISRE corresponds to STAT1/2 transcription factor binding and indicates interferon I downstream signaling. Abbreviations: WT ZNF217 wild type, KO ZNF217 knockout.

RNA-seq of *ZNF217*^*KO*^ versus *ZNF217*^*WT*^ RC-K8 lymphoma cells discovered large changes of the transcriptome with 593 upregulated and 429 downregulated genes. Results were validated in L-428 cells (Supplementary Table S8, Supplementary Fig. S2e). Global proteome analysis of RC-K8 corresponded with the RNA-seq findings (Supplementary Table S8, Fig. 3). Pre-ranked gene set enrichment analysis (GSEA) of RNA-seq data verified a significant enrichment of gene sets that have been generated upon perturbation of REST, HDAC3, and LSD1/KDM1A (Fig. 3c).

**Fig. 4:**
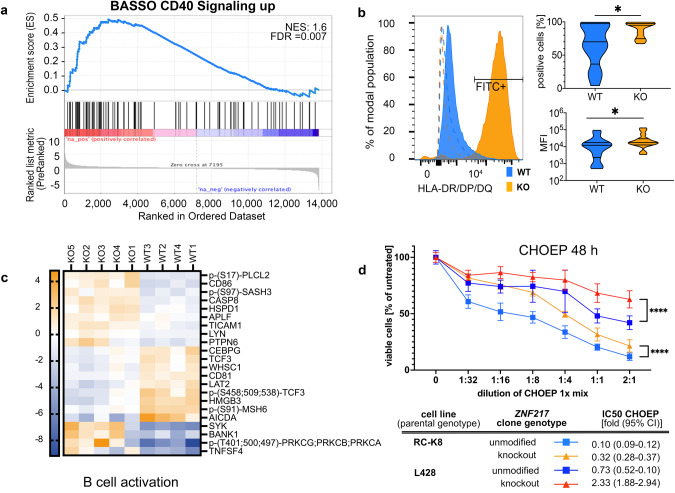
B cell activation signature after knockout of *ZNF217*. **a** Pre-ranked gene set enrichment analysis (GSEA) was performed based on a gene signature derived from CD40 activation [60]. Graph shows normalized enrichment scores (NES) and false discovery rate (FDR) *q*-values of GSEA analysis **b** Flow cytometry analysis of MHC class II presentation of ZNF217^KO^ (*n* = 15) and ZNF217^WT^ (*n* = 13; data obtained from *n* = 3 independent experiments). Left side: representative histogram of fluorescence shift comparing one anti-HLA-DR/DP/DQ-FITC-stained WT with one randomly assigned KO sample. Dashed lines: isotype controls. Right side: quantification of positive cells over threshold (top) and mean fluorescence intensity (bottom). Mann–Whitney test; *<0.05. **c** Significantly differentially abundant (phospho-)proteins (FDR < 0.05) associated with B cell activation visualized by Euclidean clustering of z-scores (Supplementary Table S8.4 and S8.5). **d** Treatment of cell clones with R-CHOEP resulted in a lower IC50 in ZNF217^WT^ versus ZNF217^KO^ cells of 3.2 to 3.4 -fold. Figure shows merged data from *n* = 11 different ZNF217^KO^ or ZNF217^WT^ clones obtained from *n* = 3 independent WST-1 assay experiments after 48 h of increasing dilutions of R-CHOEP treatment (mean with 95% CI; Mixed effect model with Geisser–Greenhouse correction). 1x R-CHOEP is defined in Supplementary Table S4. *q* indicated by *<0.05; **<0.01, ***<0.001, ****<0.0001.

Most notably, the gene expression changes upon *ZNF217* knockout demonstrated altered gene expression of type I and II interferon-downstream signals (Fig. 3d), generating expression signatures reminiscent of activated B cells, such as a CD40-signature with upregulation of CD80, CD86, OX-40L, and MHC class II genes (Figs. 3 and 4, Supplementary Table S8, Supplementary Fig. S3). These changes were evident on RNA and protein levels and validated for surface presentation of functional MHC II receptors in vitro (Fig. 4b), confirming the CD40-signature detected by Nanostring expression profiling of primary *ZNF217*- altered PMBCL samples (Supplementary Fig. S1c). Notably, a lower sensitivity to chemotherapeutic drugs (CHOEP) with ~3-fold higher relative IC50 was observed in *ZNF217*^*KO*^ cells, suggestive for a resistance mechanism caused by CD40 stimulation, which has been described in lymphoma cells before [36] (Fig. 4d).

In accordance, phospho-proteome analysis demonstrated an increased phosphorylation in proteins involved with cell adhesion, cytoskeleton organization, and GTPase activity suggesting an increased cellular turnover associated with cellular trafficking/antigen presentation and cell-cell interaction. It further identified a negative enrichment of phosphoproteins associated with chromosomal reorganization and condensation after *ZNF217* knockout (Supplementary Table S8).

**Fig. 5:**
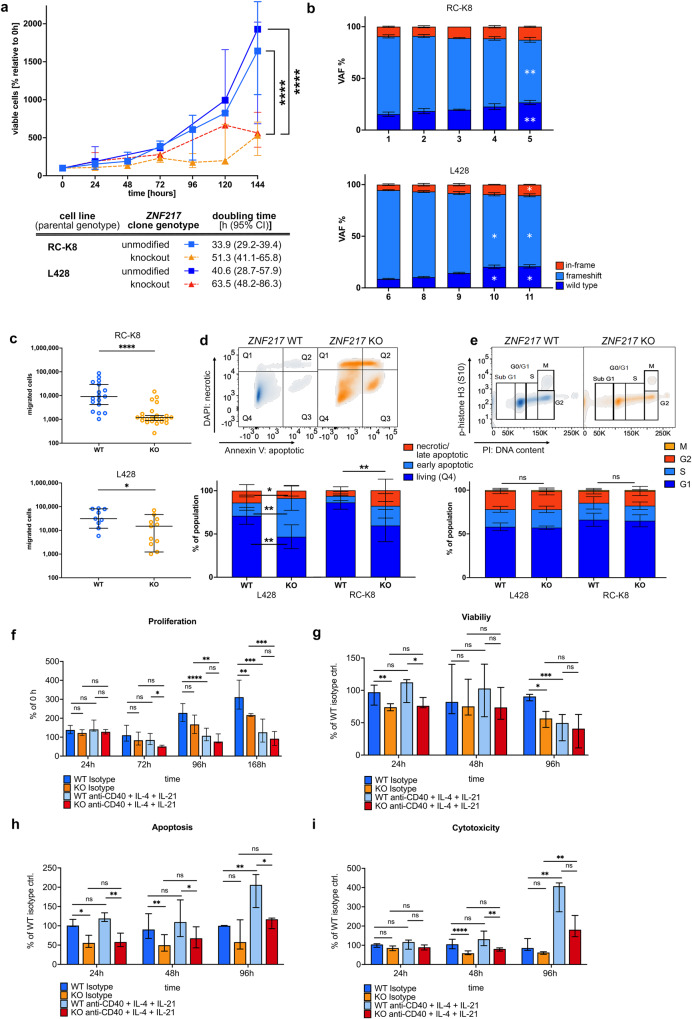
Expression of aberrant ZNF217 induced large phenotypic changes in B cell lymphoma cell lines and interfered with oncogenic signaling pathways. ZNF217^KO^ and ZNF217^WT^ single cell clones were analyzed in cell-based functional assays. **a** Baseline growth characteristics of RC-K8 (KO: *n* = 28 and WT: *n* = 19) and L-428 (KO *n* = 11 and WT *n* = 9) were determined by longitudinal WST-1 viability assays after 6 h of serum starvation. Raw data of growth curves shows median and interquartile range of merged replicates of *n* ≥ 3 independent experiments. Growth curves were fitted with an exponential (Mathusian) growth model and extra sum-of-squares *F* test was used to compare proliferation/viability. **b** Bulk cells were transfected with guide RNA and Cas9 and longitudinally analyzed in *n* = 3 independent experiments for their relative proportion of genotypes by Sanger sequencing. Time points indicate weeks after transfection. Friedman-Test and Dunn’s post test were used, bars show mean (SD). **c** ZNF217^KO^ and ZNF217^WT^ cells were analyzed for their migratory potential in a Boyden Chamber assay after 24 h. Median and 95% CI of means of biological replicates (total *n*: RC-K8: WT: *n* = 46, KO: *n* = 67, L-428: WT: *n* = 18, KO: *n* = 22) of *n* ≥ 3 independent experiments are shown. Statistical significance was determined by Mann–Whitney-Test. **d, e** Determination of apoptotic/dead cell content in passaging of ZNF217^KO^ (*n* = 14) versus ZNF217^WT^ (*n* = 8) clones using Annexin-V- and DAPI-based flow cytometry, and cell cycle analysis (WT: *n* = 14, KO: *n* = 28). Upper panel shows representative flow cytometry density plots of ZNF217^WT^ and ZNF217^KO^ examples of RC-K8. Means of *n* ≥ 3 independent experiments were merged and error bars show SD. **f** Proliferation of ZNF217^KO^ (*n* = 6) and ZNF217^WT^ (*n* = 4) clones upon stimulation in duplicates with an agonist anti-CD40 antibody, IL-4, and IL-21. One representative experiment of three independent ones (total n: KO: n = 14) and WT: *n* = 16. Bars show median and interquartile range (Welch’s ANOVA test with Dunett’s T3 multiple comparisons test). **g**–**i** ZNF217^KO^ (*n* = 9) and ZNF217^WT^ (*n* = 10) clones were treated in triplicates with anti-CD40 antibody, IL-4, and IL-21. Control cells were moved to control media after 48 h and viability, apoptosis, and cytotoxicity were measured at the indicated time points after removal. Merged data of three independent experiments, bars show median and interquartile range (Kruskal–Wallis test, Dunn’s multiple comparisons test). *q* indicated by *<0.05; **<0.01, ***<0.001, ****<0.0001.

Despite the observed CD40 activation-like signature, knockout of *ZNF217* did not trigger increased proliferation or anti-apoptosis. It rather decreased the overall proliferation rate with a~1.5-fold extended doubling time in *ZNF217*^*KO*^ compared to *ZNF217*^*WT*^ cells (Fig. 5). Co-culture of different *ZNF217* variants over a period of several weeks revealed a significant proliferative disadvantage of clones harboring *ZNF217* frameshift mutations. Furthermore, *ZNF217*^*KO*^ cells presented a decrease in migratory potential and an increased fraction of apoptotic cells during passaging without differences in cell cycle behavior. This indicates that the slower increase in viable cell count was determined by a stronger commitment to apoptosis rather than by a shorter generation time. Stimulating *ZNF217*^*KO*^ and *ZNF217*^*WT*^ cells with an agoniztic CD40 antibody and IL-4/IL-21, induced a similar effect (decreased viability and proliferation, increased apoptosis and cytotoxicity) in *ZNF217*^*WT*^ cells leveling the phenotypic differences between wild type and mutant cells over time (Fig. 5f–i). Growth-inhibitory effects of CD40 stimulation have been previously observed in several lymphoma and carcinoma cell lines in vitro [37]. The CD40 signature in primary material and in cell lines as well as the phenocopying of the effect by agonist antibody-based CD40 stimulation emphasizes a role of ZNF217 in late germinal center differentiation.

### ZNF217 interferes with CoREST- and LSD1-containing protein complexes and loss of ZNF217 increases chromatin accessibility

**Fig. 6:**
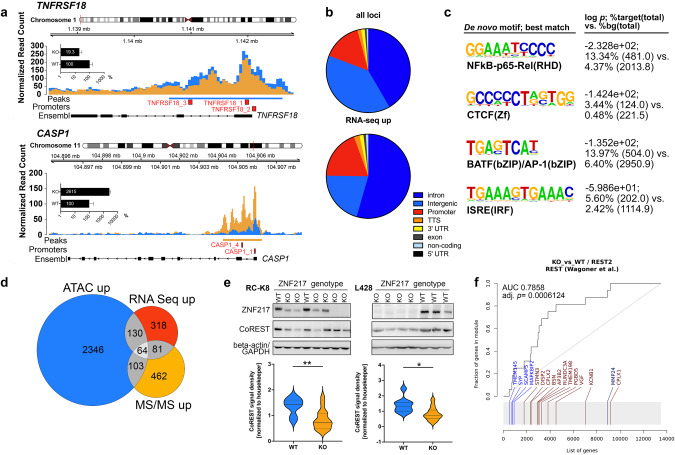
Loss of ZNF217 led to epigenetic changes by interfering with the CoREST histone modifier complex. **a** ATAC-sequencing detected an increased chromatin accessibility in ZNF217^KO^ cells (*n* = 5) compared to ZNF217^WT^ cells (*n* = 4). Nucleosome mapping revealed differentially accessible chromatin areas in intronic and around promoter regions of 194 genes, which were found differentially expressed in transcriptome analysis, including genes of the TNF and interleukin signaling (representative maps are shown for *CASP1* and *TNFRSF18*, bar graph indicates relative expression on transcript level, log scale). Red squares denote promoter regions. **b** Functional annotation of all loci with differential ATAC peaks and of all 192 loci with different chromatin accessibility and gene upregulation determined by RNA-seq. Promoter regions were defined as ± 2500 bp to nearest TSS. **c** De novo motif enrichment analysis (log *p*-value, % of targets sequences with known motif out of number of target sequences with motif is shown versus % of background sequences with known motif out of number of background sequences with motif is shown; four top hits ranked by *p*-values are depicted; Supplementary Table S8). **d** A number of 64 genes were differentially upregulated in all three multi-omics layers (Supplementary Table S8). **e** Western blot analysis of ZNF217^WT^ (RC-K8: *n* = 8; L-428: *n* = 9) and ZNF217^KO^ (RC-K8: *n* = 15; L-418: *n* = 12). Representative blots of one out of three independent experiments and densitometric quantification of all replicates are shown. **f** Enrichment plot of gene expression data from RNA-sequencing demonstrating a significant enrichment of a gene signature resulting from a perturbation of RE1 Silencing Transcription Factor (REST). Visualization was performed using the tmod R package, (light) blue indicates (significant) downregulation, (light) red indicates (significant) upregulation.

As shown by ATAC-seq, knockout of *ZNF217* increased the accessibility of genomic areas in 86% of the differentially accessible loci and affected predominantly intronic (42%), intergenic (39%) and promoter regions (15%). Among 194 genes concomitantly upregulated in RNA-seq and differentially accessible in ATAC-seq, 54% and 21% were affected at intronic and intergenic regions, respectively, and 19% at promoter regions (Fig. 6a–d, Supplementary Table S8). This suggests a role of ZNF217 in functional regulation of gene expression at promoters and distal cis-regulatory regions predominantly resulting in a de-repression of target gene expression upon knockdown. Unbiased de novo motif analysis identified a number of enriched motifs in open chromatin areas, which were consensus for major transcription factors, such as NF-κB, BATF family, and interferon-sensitive response elements, such as IRFs (Fig. 6c, Supplementary Table S8). Importantly, an increased chromatin accessibility to consensus binding sequences for CTCF, a major regulator of chromatin architecture, was discovered.

**Fig. 7:**
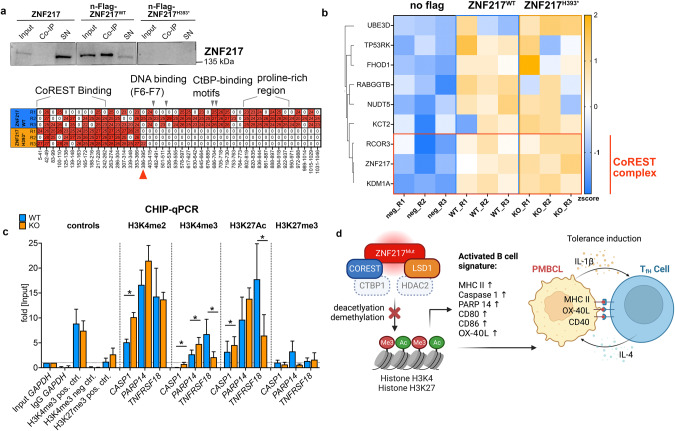
Interaction partner of ZNF217 in lymphoma cells. **a** Western blot of anti-flag immunoprecipitated protein complexes after ectopic expression of *n*-Flag-ZNF217 (*n* = 3) or the n-Flag-ZNF217^H393*^ (*n* = 3) variant in ZNF217^KO^ cells (Supplementary Fig. S4). The samples with the truncated protein variant did not show any western blot signal (top), since the antibody recognizes a c-terminal epitope. However, peptides of amino acids 1-393 were detected in the mass spectrometry analysis (bottom). **b** Co-immunoprecipitated proteins after anti-flag pulldown of *n* = 3 biological replicates in two repeated experiments, data of one experiment is shown; neg R1-R2: no-Flag ZNF217^WT^ controls, R1-R3: *n*-Flag-ZNF217^WT^ and R1-R3: *n*-Flag-ZNF217^H393*^ were analyzed by mass spectrometry-based proteomics and included 2777 proteins for quantitation (filter: min 3 valid values in at least one group). LFQ intensities were used for analyses, with imputation. All variants were compared to no-Flag-tag control. Enriched proteins (FDR 20%, or log_2_fold change >2, -log_10_ *p* > 1.3 (0.05)) were defined as ZNF217 binders. A total of 9 binders were identified for wild type and truncated ZNF217, of which 3 known members of the CoREST complex clustered together (Euclidean distance): ZNF217, CoREST 3 and LSD1/KDM1A. Heatmap shows z score-transformed LFQ intensities. **c** Chromatin-Immunoprecipitation- (CHIP-) *q*PCR analysis of major target histone marks affecting differentially expressed genes. Target areas for PCR primers were determined from ATAC-sequencing data, *q*PCR was quantified using the percent input method. Graph shows mean (SD), *q < 0.05; multiple paired t-tests corrected for multiple testing (FDR) of *n* = 4 ZNF217^WT^ and *n* = 4 ZNF217^KO^ clones from *n* ≥ 3 independent experiments. MS/MS: tandem mass spectrometry. FDR indicated by *<0.05; **<0.01. **d** Summary: Proposed role of ZNF217 in PMBCL: Depiction of the protein complex, including LSD1, CoREST, ZNF217 and most likely HDAC2 and CtBP1, which is perturbed by mutation of ZNF217 and thereby affects H3K4 methylation and H3K27 acetylation leading to altered expression of genes involved in B cell differentiation activation. Upregulation of crucial antigens involved in T cell-interaction suggests a tolerance-induction process. Illustration was created with BioRender.com.

Mass spectrometry, RNA-seq, and Western blot results suggest that *ZNF217* perturbation interferes with the activity of a CoREST-containing silencing complex by showing (i) decreased expression of CoREST in *ZNF217*^*KO*^ cells (Fig. 3b, e), (ii) gene signatures that resemble REST [38], HDAC, and LSD1 perturbation (Figs. 3c, 6f), and (iii) a significant co-enrichment of CoREST3 and LSD1 (*KDM1A*) upon ectopic expression of a ZNF217-Flag fusion protein, pulldown and binding partner analysis (Fig. 7a,b, Supplementary Fig. S4). Notably, other CoREST complex partners (HDAC2 and CtBP1) were detected as well, albeit, with non-significant enrichment (Supplementary Table S8, Supplementary Fig. S4b).

We found decreased RCOR1 protein levels without altered mRNA or chromatin accessibility, indicating that ZNF217 might be involved in the stabilization of CoREST family proteins and/or their complexes. In fact, an ectopically expressed *ZNF217*^H393*^ variant did not show a differential binding of identified CoREST complex partners, but an enrichment of an E3 ubiquitin-protein ligase (UBE3D) in the H393* pulldown (Supplementary Fig. S4). Enrichment of ZNF217 in proteasome inhibitor-treated *ZNF217*^*KO*^ cells, demonstrated proteasomal degradation of translated ZNF217 variants (Supplementary Fig. S4d). Large changes in the abundance of (phospho-) proteins of the ubiquitin proteasome system upon knockout of *ZNF217* underlined these findings (Supplementary Fig. S4e). Since ubiquitination regulates proteasomal degradation, loss of *ZNF217* might influence the stability of CoREST/ZNF217-containing chromatin modifier complexes and exhibit non-catalytic activity in CoREST complex guidance.

Finally, we analyzed the histone modification pattern in representative differentially accessible regions of target genes (promoter-/intron 1-regions of *CASP1* and *TNFRSF18*, and 3’UTR/TSS region of *PARP14*) in *ZNF217*^*KO*^ and *ZNF217*^*WT*^ cells. To analyze major known target histone marks of ZNF217 [39], LSD1, and HDAC2, we determined H3K4 methylation and H3K27 acetylation [33], as well as a putative negative histone mark, H3K27me3, which is primarily affected by non-interacting EZH2/PRC2. CHIP-qPCR analysis demonstrated that *ZNF217*^*KO*^ significantly altered H3K4me2, H3K4me3, and H3K27Ac in accordance with differential expression, while it did not affect H3K27me3 in any of these three genes (Fig. 7c). This gives further evidence that *ZNF217* is interacting in a CoREST/LSD1/HDAC-containing complex. Disruption of *ZNF217* induces gene expression changes associated with B cell differentiation and activation by altering their epigenetic state (Fig. 7).

## Discussion

Little is known about the function of ZNF217 in cancer today. In metastatic cancer, *ZNF217* amplification ranks among the most frequent genetic alterations and its overexpression was reported to be associated with poor outcome, suggesting an oncogenic function [21, 40, 41]. In contrast, we show that *ZNF217* aberrations in PMBCL often display as deletions and/or truncating frameshift alterations spread all over the coding region suggestive of tumor suppressor properties. This opposing pattern of genetic aberrations between solid and lymphoid malignancies also suggests tissue-specific functions of ZNF217.

We identified several upregulated genes in *ZNF217-*deficient primary material, which support the hypothesis of ZNF217 being involved in aberrant B cell differentiation and impaired immune surveillance. TSLP, which is expressed in thymic stromal cells, regulates the development of B cells. It was strongly upregulated in the *ZNF217*-deficient primary PMBCL samples. Due to the lack of stromal cells in the cell culture, no analogous effect could be detected in vitro. However, local expression of TSLP directly stimulates pre–B cell proliferation and systemic expression of TSLP in mice has been shown to interfere with B lineage-committed hematopoiesis [42].

Using lymphoma cell lines, we demonstrate that ZNF217 interacts with chromatin modifying proteins, most notably LSD1 and CoREST. Disruption of the interaction led to changes in histone marks that have been assigned to repressive chromatin states [33, 43]. Some of these chromatin modifiers have also been shown to play fundamental roles in B cell development [13, 14].

By increasing pro-apoptotic priming, antigen presentation and altering NF-κB- and interferon-downstream signaling, in vitro *ZNF217* knockout interfered with B cell differentiation processes and enriched phenotypes that are usually associated with B cell activation and maturation [44]. The functional pattern of *ZNF217* loss in PMBCL in vitro resembles a phenotypic switch between naïve B cell properties and light zone properties of GC B cells [44, 45]. This reflects the data of primary material, where interferon and cytokine signaling was altered in patients with *ZNF217* aberrations showing expression patterns, remarkably distinct from expression changes induced by other frequently altered chromatin remodelers. However, it should be considered that a bi-allelic *ZNF217* knockout model was used to understand the general function of ZNF217 in high grade lymphomas, but might not fully mirror the effect of heterozygous point mutations, which did not lead to decreased *ZNF217* transcript levels in our primary cohort.

The upregulation of *CASP1* and *PARP14* and the downregulation of *TNFRSF18*, on which we followed up for technical validation of the complex function, underlined the functional connection of *ZNF217* with B cell activation. PARP14 has been described to function as a transcriptional switch upon IL-4 stimulation, which is vital for fine tuning CD40 ligand-induced B cell proliferation, cell survival, and division-linked isotype switching together with IFN-γ [46]. In addition, Caspase 1 activates IL-1, which, after secretion, triggers expression of CD40L on T cells and release of IL-4 from T follicular helper cells [47]. It thereby regulates the germinal center-associated cellular crosstalk. GC B cells have shown lower GITR expression levels in murine B cell cultures stimulated with anti-CD40 antibodies, IL-4, IFNγ, or IFNα [48].

Most notably, the activation of interleukin- and interferon-α and -γ signaling as common mechanisms identified in both, primary *ZNF217*-deficient samples and after knockout in vitro strongly supports an immunomodulatory effect of *ZNF217* loss. B cells treated with IFN-α and -β have been shown to present an increased survival and resistance to Fas-mediated apoptosis [49]. In addition, IFN-γ is able to boost immune escape by induction of PD-L1, PD-L2, and IDO1 expression [50, 51]. We found IDO1/Kynurenine strongly upregulated on RNA and protein level.

The CD40 signature identified in vitro and in primary material provides evidence for a possible pathomechanism, since sustained CD40 activation can trigger lymphomagenesis and immune evasion [52]. Especially in thymic B cells, which are most probably the cells of origin of PMBCL, CD40 stimulation licenses upregulation of CD80, CD86 and MHC class II receptors. This process shows striking resemblance to the GC reaction and ultimately drives self-tolerance of neoantigens in thymocytes by both clonal deletion and Treg differentiation [53, 54].

On a molecular level, truncation of *ZNF217* did not lead to altered binding of complex partners, but to changes in the modification patterns of target histone marks of HDACs and LSD1, affecting the accessibility for transcription factors. Motif analysis identified particularly binding motifs of the NF-κB, BATF/AP1, and IRF family of proteins, which are transcription factors crucial for B cell maturation and lymphomagenesis. ZNF217 loss also altered the accessibility for consensus motifs of CTCF, a major regulator of global 3D chromatin architecture. In this regard, lessons may be learned from the recent findings about histone H1 loss, which acts as a bona fide tumor suppressor and drives malignant transformation primarily through three-dimensional genome reorganization [11]. Since our data suggests that *ZNF217* influences the stability of CoREST/ZNF217-containing chromatin modifier complexes, it likely exhibits non-catalytic activity in complex guidance where it interferes with B cell differentiation-associated patterns of chromatin structure.

However, there are limitations of the study to consider. Despite the high prevalence of *ZNF217* mutations, a clear tumor suppressive function of *ZNF217* could not be demonstrated. Contradictive findings include the lack of downregulated *ZNF217* transcript levels in patient samples and lack of cell-cycle regulatory, pro-apoptotic or anti-proliferative effects in vitro. This indicates complex functions of *ZNF217* in the context of additional aberrations and immune interactions, which require more sophisticated model systems, for example an in vivo model, to be fully deciphered.

Concluding, our study provides evidence that disruptive mutations of *ZNF217* frequently found in aggressive lymphomas interfere with histone modifications at genes that control major hallmarks of lymphomagenesis such as cytokine signaling, B cell differentiation and interactions within the immune cell compartment.

### Supplementary information

Supplementary data file

Supplementary Table S1

Supplementary Table S2

Supplementary Table S8

## Data Availability

The mass spectrometry proteomics data have been deposited to the ProteomeXchange Consortium via the PRIDE partner repository with the dataset identifiers PXD034337 and PXD040928. RNA-seq and ATAC-seq reads have been deposited to the Sequence Read Archive (SRA) and are accessible via BioProject PRJNA851197.
