# Supplementary material for: Frequent ZNF217 mutations lead to transcriptional deregulation of interferon signal transduction via altered chromatin accessibility in B cell lymphoma
Source: Leukemia. 2023 Aug 30;37(11):2237–49. doi: 10.1038/s41375-023-02013-9 (PMC10624633; doi:10.1038/s41375-023-02013-9)
Supplement: Supplementary file 1 — Supplementary data file [file 41375_2023_2013_MOESM1_ESM.pdf]

Supplementary Information

Frequent *ZNF217* mutations lead to transcriptional deregulation of interferon signal transduction via altered chromatin accessibility in B cell lymphoma

Franziska Briest[1#], Daniel Noerenberg[1#], Cornelius Hennch[1#], Kenichi Yoshida[2,3], Raphael Hablesreiter[1], Jose Nimo[1], Daniel Sasca[4], Marieluise Kirchner[5], Larry Mansouri[6], Yoshikage Inoue[2], Laura Wiegand[1], Annette M. Staiger[7,8], Beatrice Casadei[9], Penelope Korkolopoulou[10], January Weiner[11], Armando Lopez-Guillermo[12], Arne Warth[13], Tamás Schneider[14], Ákos Nagy[15], Wolfram Klapper[16], Michael Hummel[17,18], George Kanellis[19], Ioannis Anagnostopoulos[17,20], Philipp Mertins[5], Lars Bullinger[1,18], Richard Rosenquist[6,21], Theodoros P. Vassilakopoulos[22], German Ott[7], Seishi Ogawa[2,23,24] and Frederik Damm[1,18]

[1]Department of Hematology, Oncology and Cancer Immunology, Campus Virchow, Charité – Universitätsmedizin Berlin, corporate member of Freie Universität Berlin and Humboldt-Universität zu Berlin, Berlin, Germany.
[2]Department of Pathology and Tumor Biology, Graduate School of Medicine, Kyoto University, Kyoto, Japan.
[3]Cancer Genome Project Wellcome Trust Sanger Institute, Hinxton, United Kingdom.
[4]Department of Hematology, Oncology, and Pulmonary Medicine, University Medical Center, Johannes Gutenberg-University, Mainz, Germany.
[5]Core Unit Proteomics, Berlin Institute of Health at Charité - Universitätsmedizin Berlin and Max-Delbrück-Center for Molecular Medicine, Berlin, Germany.
[6]Department of Molecular Medicine and Surgery, Karolinska Institutet, Stockholm, Sweden.
[7]Department of Clinical Pathology, Robert-Bosch-Krankenhaus, Stuttgart, Germany.
[8]Dr Margarete Fischer-Bosch Institute of Clinical Pharmacology Stuttgart, and University of Tuebingen, Stuttgart, Germany.
[9]IRCCS Azienda Ospedaliero-Universitaria di Bologna, Istituto di Ematologia “Seràgnoli”, Bologna, Italy.
[10]First Department of Pathology, National and Kapodistrian University of Athens, Laikon General Hospital, Athens, Greece.
Core Unit Bioinformatics Berlin, Berlin Institute of Health at Charité - Universitätsmedizin Berlin, Berlin, Germany.
Hematology Department, Hospital Clinic, University of Barcelona, Barcelona, Spain
[11]Core Unit Bioinformatics Berlin, Berlin Institute of Health at Charité - Universitätsmedizin Berlin, Berlin, Germany.
[12]Hematology Department, Hospital Clinic, University of Barcelona, Barcelona, Spain
[13]Institute of Pathology, University Hospital Heidelberg, Heidelberg, Germany.
[14]National Institute of Oncology, Budapest, Hungary.
[15]HCEMM-SE Molecular Oncohematology Research Group, Department of Pathology and Experimental Cancer Research, Semmelweis University, Budapest, Hungary.
[16]Department of Pathology, Hematopathology Section and Lymph Node Registry, Universitätsklinikum Schleswig-Holstein, Kiel, Germany.
[17]Department of Pathology, Charité – Universitätsmedizin Berlin, corporate member of Freie Universität Berlin and Humboldt-Universität zu Berlin, Berlin, Germany.
[18]German Cancer Consortium (DKTK) and German Cancer Research Center (DKFZ), Heidelberg, Germany.
[19]Department of Hematopathology, Evangelismos General Hospital, Athens, Greece.
[20]Institute of Pathology, University of Würzburg and Comprehensive Cancer Center (CCC) Mainfranken, Würzburg, Germany.
[21]Clinical Genetics, Karolinska University Hospital, Stockholm, Sweden.
[22]Department of Hematology and Bone Marrow Transplantation, National and Kapodistrian University of Athens, Laikon General Hospital, Athens, Greece.
[23]Institute for the Advanced Study of Human Biology (WPI-ASHBi), Kyoto University, Kyoto, Japan.
[24]Department of Medicine, Centre for Haematology and Regenerative Medicine, Karolinska Institutet, Stockholm, Sweden.

# F.B., D.N. and C.H. contributed equally as first authors

Table of contents

- Supplementary Figures
  - Supplementary Figure S1. Cancer plot of ZNF217 alterations in PMBCL patients and expression patterns upon genetic alterations in frequently altered epigenetic modifiers in PMBCL.
  - Supplementary Figure S2. Generation of ZNF217 altered cell clones.
  - Supplementary Figure S3: Differentially abundant (phospho-)proteins in ZNF217WT and ZNF217KO cells.
  - Supplementary Figure S4. Enrichment of ZNF217-binding proteins in ZNF217WT and ZNF217KO pulldown samples.
- Supplementary Tables
  - Supplementary Table S1. Sample summary.
  - Supplementary Table S2. Primary PMBCL results.
  - Supplementary Table S3. Additional probes included in the Nanostring Panel.
  - Supplementary Table S4. Stock concentration ratios of cytostatic agents applied in vitro
  - Supplementary Table S5. sgRNAs used for knockout of ZNF217
  - Supplementary Table S6. Primers for ATAC library preparation
  - Supplementary Table S7. Primers used for CHIP-qPCR
  - Supplementary Table S8. In vitro multi-omics data.
- Supplementary Methods
  - Gene panel design
  - Target sequencing
  - RNA Profiling on Nanostring nCounter
  - Transfection of human cell lines
  - Flow Cytometry
  - RNA Sequencing
  - Mass-Spectrometry LC-MS/MS
  - Cloning and Mutagenesis
  - Assay for Transposase-Accessible Chromatin with high- throughput sequencing (ATAC-Seq)
  - Co-immunoprecipitation and ZNF217 interactome analyses by mass spectrometry
  - Chromatin-immunoprecipitation followed by quantitative PCR (CHIP-qPCR)
  - Data analysis and statistics

Supplementary Figures

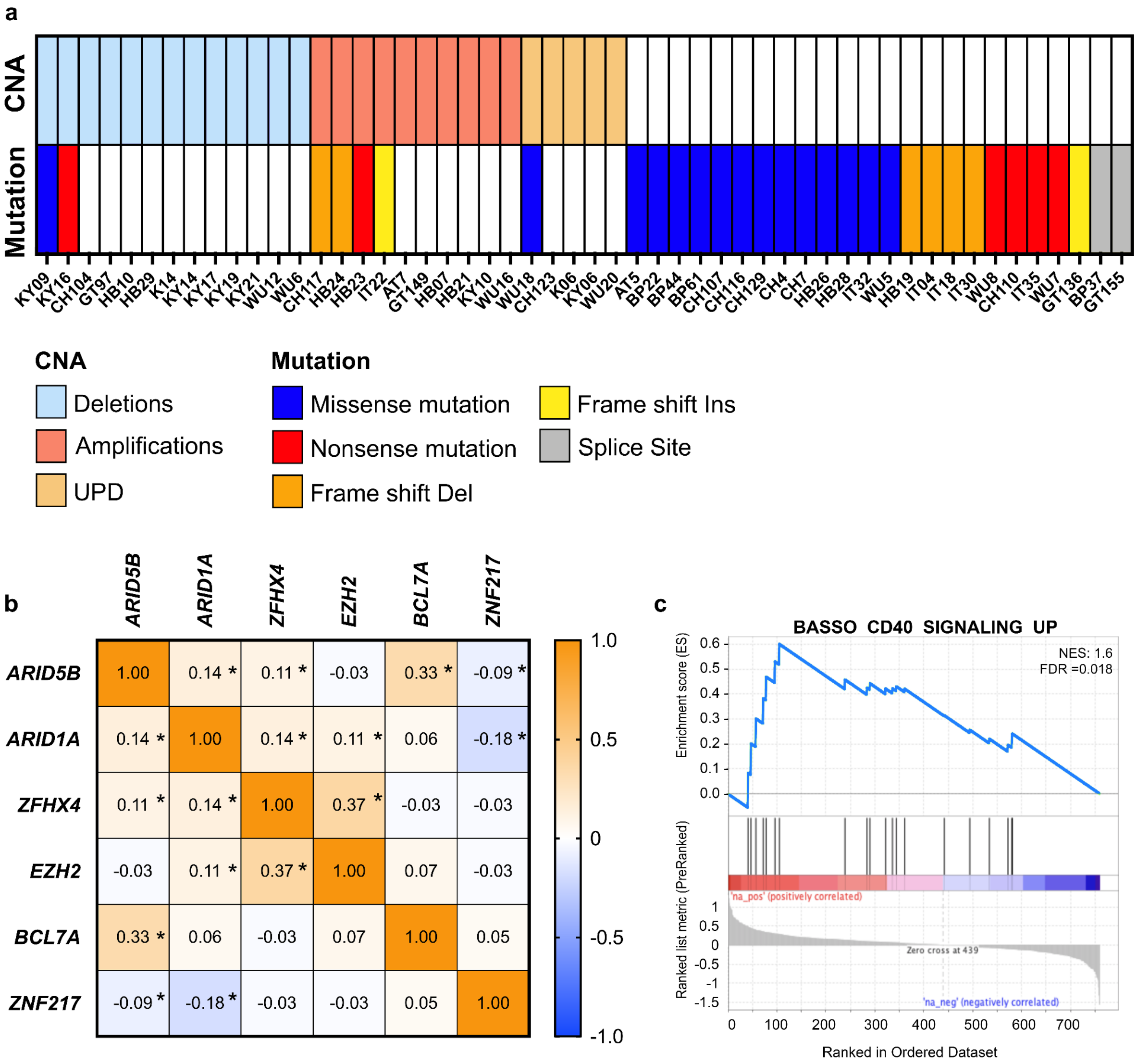

Supplementary Figure S1. Cancer plot of *ZNF217* alterations in PMBCL patients and expression patterns upon genetic alterations in frequently altered epigenetic modifiers in PMBCL.

a Distribution of *ZNF217* copy number alterations and mutations in 52 *ZNF217*-altered primary PMBCLs. In case of multiple mutations per patient, the most deleterious type was attributed to the patient. **b** Correlation matrix of expression patterns in primary material with either ≥1 copy number changes and/or ≥1 SNV in either *ARID5B, ARID1A, ZFHX4, EZH2, BCL7A,* or *ZNF217* versus respective wild types. Heatmap shows Pearson correlation coefficient. Two tailed comparisons were FDR corrected (Benjamini-Hochberg method). FDR<0.05 indicated by *. **c** *Pre-ranked gene set enrichment analysis (GSEA) of the Nanostring nCounter results was performed based on a gene signature derived from CD40 activation*[1]*. Graph shows normalized enrichment scores (NES) and false discovery rate (FDR) q-values of GSEA analysis.*

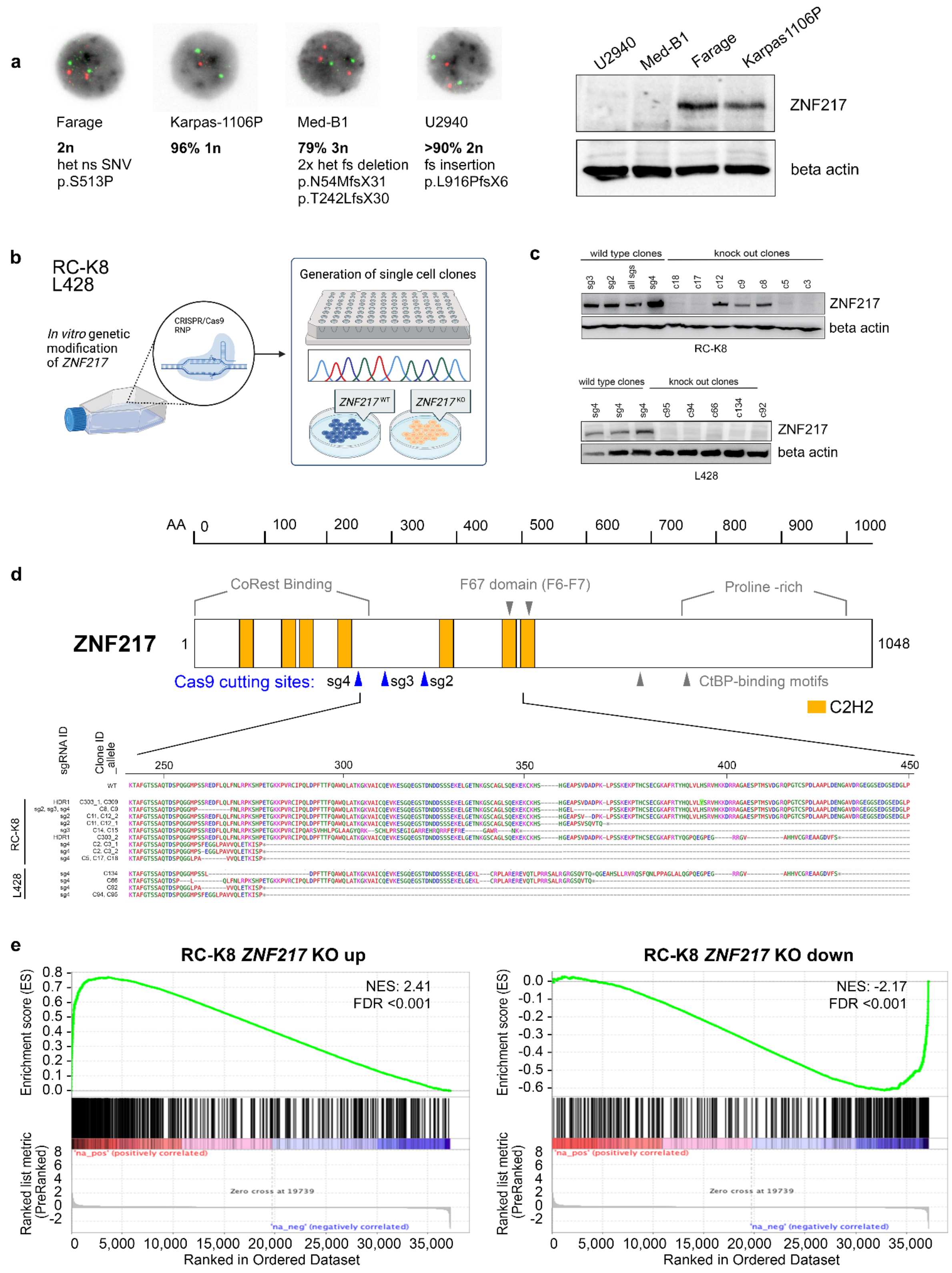

Supplementary Figure S2. Generation of *ZNF217* altered cell clones.

a Four PMBCL cell lines were excluded from the study since none of four exhibited two unimpaired alleles of *ZNF217*. FISH identified aberrant copy numbers of *ZNF217* in 2 out of 4 cell lines (red), which were validated by sequencing and copy number analysis. Sequencing also identified mutations in one or more alleles in 3 out of 4

cell lines, leading to a loss of full length protein expression in MedB-1 and U2940 cells. **b** After transfection with Cas9/sgRNA RNP targeting exome 2 of *ZNF217*, RC-K8 and L428 cells were single cell diluted and obtained monoclonal wild type and knockout cells were genotyped and analyzed by various functional assays. **c** Genetic modifications generated complete loss, truncated and full-length protein variants with in-frame alterations, as detectable with a monoclonal antibody against a c-terminal epitope of ZNF217. Clones with two altered alleles were included into the study. **d** Schematic depiction of ZNF217 and relevant protein domains. Zink-finger (C2H2) domains were marked in yellow. Cutting sites for Cas9 and the specific guide RNAs are shown. The resulting protein sequences of the generated clones was translated by use of Benchling biology software (https://benchling.com) and blasted with the Multiple Sequence Alignment tool Clustal Omega (https://www.ebi.ac.uk/Tools/msa/clustalo/) to visualize changes (access date 25.03.2020). _1 and _2 indicate specific alleles. **e** GSEA analysis of differentially expressed genes after *ZNF217* knockout (KO: n=5, WT: n=3in L428 cells were analyzed for the signatures of 593 up- or 429 down-regulated genes in RC-K8 cells after *ZNF217* knockout. *Illustration was partially created with BioRender.com*

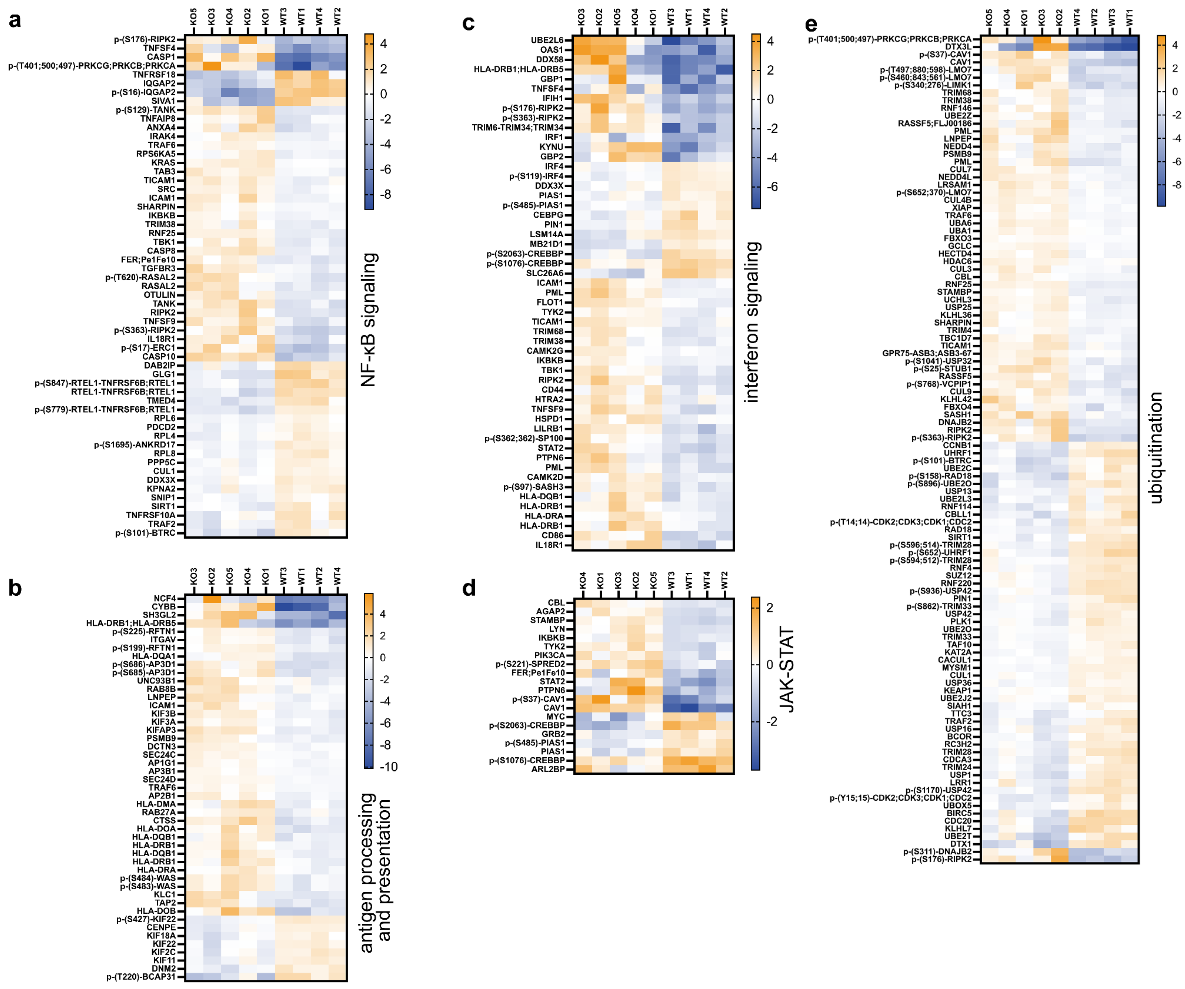

Supplementary Figure S3: Differentially abundant (phospho-)proteins in ZNF217^{WT} and ZNF217^{KO} cells.

a-e Significantly differentially abundant (phospho-)proteins (FDR<0.05) were assigned to pathways/mechanisms by keyword filtering based on ontology terms and visualized by Euclidean clustering (Suppl. Table S8.4 and S8.5).

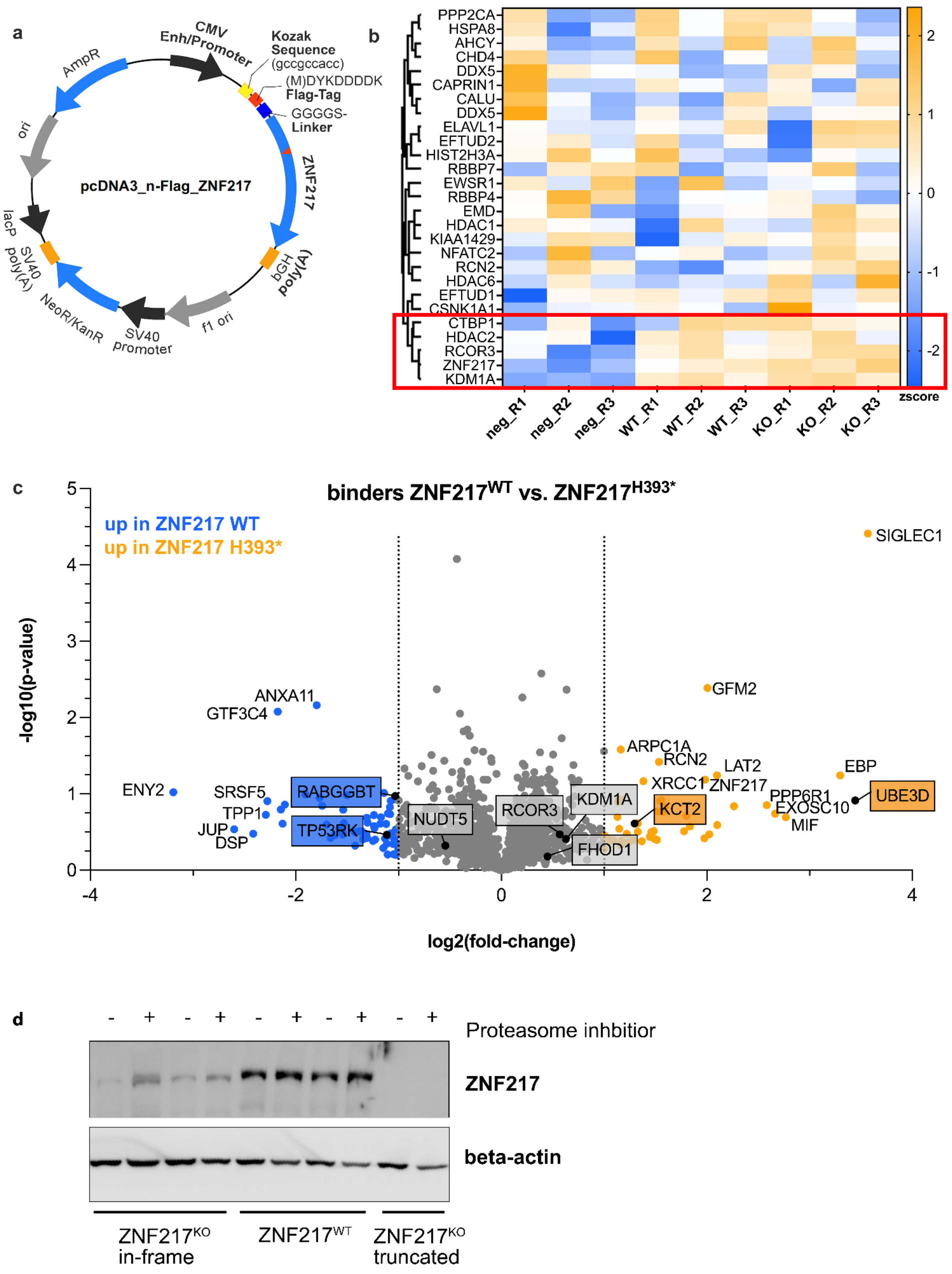

Supplementary Figure S4. Enrichment of ZNF217-binding proteins in ZNF217^{WT} and ZNF217^{KO} pulldown samples.

a Vector map of the ZNF217 overexpression vector containing a n-terminal Flag-tag and either wild type ZNF217 or a nonsense codon at amino acid 393. **b** Co-immunoprecipitated proteins after anti-flag pulldown of n=3

biological replicates (Negative controls (R1-R2): ZNF217^{WT}, WT (R1-R3): -Flag-ZNF217^{WT} and KO (R1-R3): n-Flag-ZNF217^{H393*}) were analyzed by gradient mass spectrometry. Euclidean distance clustering of known ZNF217 binders irrespective of significant enrichment (String, BioGrid database) co-clustered 5 members of a CoREST repressor complex. Heatmaps shows zscore-transformed LFQ intensities. **c** Comparison of proteins that exclusively bind to either wild type or truncated ZNF217 identified two significantly altered binders: UBE3D and RABGGTB (FDR 30%). Previously identified binders are marked in the volcano plot (excluding ZNF217). **d** Cells were treated with 10 µM bortezomib for 90 minutes to enrich for ubiquitinated proteins. Proteins were visualized by western blotting.

Supplementary Tables

Supplementary Tables S1, S2, and S8 are provided as separate excel files.

Supplementary Table S1. Sample summary.

Table S1.1. A summary of each sample that passed sequencing quality exclusion criteria for WGS, WES and/or TS. The summary includes information about ID, gender, age, country, material (Fresh Frozen, FF vs. Formalin-Fixed Paraffin-Embedded, FFPE tissue), location of the biopsy, tumor purity estimated via histology or via allele-frequency-based imputation[2], analysis method (TS: targeted sequencing, Nanostring) and DS_{200} values for the RNA.

Supplementary Table S2. Primary PMBCL results.

Table S2.1. List of 106 genes of interest included in the target re-sequencing panel **Table S2.2.** List of SNP probes **Table S2.3.** Additional probes for improved performance and focal SCNA detection **Table S2.4.** QC metrics of all samples sequenced via target sequencing (n = 157) **Table S2.5.** Curated *ZNF217* mutations (n = 57) identified in 157 PMBCL samples via target sequencing **Table S2.6.** *ZNF217* SCNA results **Table S2.7.** Pairwise associations between *ZNF217* mutations and mutations/copy number changes in relevant PMBCL altered genes. **S2.8.** Differential expression results of Nanostring RNA Panel genes.

Supplementary Table S3. Additional probes included in the Nanostring Panel.

HUGO Gene	Probe NSID
AURKA	NM_003600.2:405
CALML4	NM_033429.2:3430
CD274	NM_014143.3:49
CELA3B	XM_011541132.1:876
CIITA	NM_000246.3:470
CLPS	NM_001252598.1:172
CPA2	NM_001869.2:232
CPB1	NM_001871.2:866
CTRB1	NM_001906.4:693
ERBB3	NM_001005915.1:420
ERBB4	NM_001042599.1:7300
HNF4G	NM_004133.3:2195
IGLC3	ENST00000390325.1:176
IGLL5	NM_001178126.1:958
ITPKB	NM_002221.3:78
LRRC66	NM_001024611.1:1660
MAP2K7	NM_145185.2:495
NFKBIE	NM_004556.2:1115
NPSR1	NM_207172.1:1006
PDCD1LG2	NM_025239.3:235
PRSS1	NM_002769.4:360
S100A4	NM_002961.2:263

SMPD3	NM_018667.3:472
STAT6	NM_003153.3:2030
TGFA	NM_003236.2:780
TGFBR1	NM_004612.2:1255
TMSB4X	NM_021109.3:0
ZFP36L1	NM_004926.2:2766
ZNF217	NM_006526.2:5
ZNF217	NM_006526.2:5094

Supplementary Table S4. Stock concentration ratios of cytostatic agents applied *in vitro*

1x CHOEP	final mass conc. [mg/ml]	ratio (mass)	final conc. [µM]
Etoposide	0.00056	70	0.95
Prednisolon	0.00056	70	1.55
Vincristine	0.000008	1	0.01
Cyclophosphamide	0.004	500	15.32
Rituximab	0.002136	267	0.06
Doxorubicin	0.00028	35	0.52

Supplementary Table S5. sgRNAs used for knockout of *ZNF217*

sgRNA	sgRNA sequence	Recognition sequence
**2**	5’ GGAAGCGCCCTCCGTGGACG 3‘	5’ CGAAGCGCCCTCCGTGGACGCGG 3’
**3**	5’ GGGTGAACGGATCGAGCTGA 3‘	5’ TGGTGAACGGATCGAGCTGAGGG 3’
**4**	5’ GGGAGGAATGCCGTCCTCGA 3‘	5 AGGAGGAATGCCGTCCTCGAGGG 3’

Supplementary Table S6. Primers for ATAC library preparation

Adapter bases	Primer sequence
Ad1_noMX	AATGATACGGCGACCACCGAGATCTACACTCGTCGGCAGCGTCAGATGTG
Ad2.1_TAAGGCGA	CAAGCAGAAGACGGCATACGAGATTCGCCTTAGTCTCGTGGGCTCGGAGATGT
Ad2.2_CGTACTAG	CAAGCAGAAGACGGCATACGAGATCTAGTACGGTCTCGTGGGCTCGGAGATGT
Ad2.4_TCCTGAGC	CAAGCAGAAGACGGCATACGAGATGCTCAGGAGTCTCGTGGGCTCGGAGATGT
Ad2.5_GGACTCCT	CAAGCAGAAGACGGCATACGAGATAGGAGTCCGTCTCGTGGGCTCGGAGATGT
Ad2.6_TAGGCATG	CAAGCAGAAGACGGCATACGAGATCATGCCTAGTCTCGTGGGCTCGGAGATGT
Ad2.7_CTCTCTAC	CAAGCAGAAGACGGCATACGAGATGTAGAGAGGTCTCGTGGGCTCGGAGATGT
Ad2.8_CAGAGAGG	CAAGCAGAAGACGGCATACGAGATCCTCTCTGGTCTCGTGGGCTCGGAGATGT
Ad2.9_GCTACGCT	CAAGCAGAAGACGGCATACGAGATAGCGTAGCGTCTCGTGGGCTCGGAGATGT
Ad2.10_CGAGGCTG	CAAGCAGAAGACGGCATACGAGATCAGCCTCGGTCTCGTGGGCTCGGAGATGT
Ad2.11_AAGAGGCA	CAAGCAGAAGACGGCATACGAGATTGCCTCTTGTCTCGTGGGCTCGGAGATGT

Supplementary Table S7. Primers used for CHIP-qPCR

Target/Primer name	Primer sequence
*CASP1*_Intron1_Rv	TTCCAGGGACTGATGTGGAAA
*CASP1*_Intron1_Fwd	TGTCCCCCTCCTCACAGTTG

*MYT1*_Exon12_Rv	GGCCTGACTGCAAATTCCAC
*MYT1*_Exon12_Fwd	CCACTGCCATCCTGAACCTC
*PARP14*_TSS_Rv	CCACAGCCAAGGCCAAAGTA
*PARP14*_TSS_Fwd	GGAGTTAGGAGGGGGTGTGG
*TNFRSF18*_Intron1_Rv	CTGGGGATGAGGGTTCAGC
*TNFRSF18*_Intron1_Fwd	ACAGAGCCTCCCTTCCCAAC
*HOXA9*_TSS_Fwd	GTGCCACCAAGTTGTTACATG
*HOXA9*_TSS_Rv	CAGGAACGAGTCCACGTAGT
*GAPDH*_TSS_Rv	GGGGAAGGGACTGAGATTGG
*GAPDH*_TSS_Fwd	GTAAGGGTCCCCGTCCTTGA

Supplementary Table S8. *In vitro* multi-omics data.

Table S8.1. differentially expressed genes KO vs. WT *ZNF217* RC-K8 **Table S8.2.** differentially expressed genes KO vs. WT *ZNF217* L428 **Table S8.3** Preranked GSEA analysis top enriched gene sets **Table S8.4.** Global proteome data KO vs. WT *ZNF217* in RC-K8 (filtered 2 min peptides) **Table S8.5.** Phospho-proteome data KO vs. WT *ZNF217* in RC-K8 (filtered 2 min MSMS) **Table S8.6** STRING-based[3] enrichment analysis of phospho-proteome data: Biological Process (GO). **S8.7** ATAC-Seq differentially accessible regions annotated Hg19, KO vs WT *ZNF217* in RC-K8 **Table S8.8.** Gene signatures associated with REST loss[4,5] used for Enrichment analysis of RNA Seq data KO vs. WT *ZNF217* RC-K8 **Table S8.9.** differentially expressed/accessible genes/proteins, concordant in MS/MS, RNA-Seq and ATAC-Seq of ZNF217 knockout RC-K8 single cell clones, related to wild type single cell clones *in vitro.* **Table S8.10. Homer *de novo* motif results.** Motifs with significant increase in accessibility are shown. **Table S8.11** Differentially binding proteins at WT or truncated ZNF217 identified by mass spectrometry after ectopic expression of either ZNF217 or the ZNF217 H393* variant in RC-K8 cells.

Supplementary Methods

Gene panel design

A designated panel of 106 genes was selected to generate a custom cRNA bait library (SureSelect, Agilent Technologies), which was used for targeted gene capture. Genes were included for either of the following reasons (Suppl. Table S2):

(i) driver genes identified via *MutSig2CV* and *dNdScv* with a q-value <0.01 based on preliminary results of 22 whole-exome sequenced cases (n=34)

(ii) recurrently mutated genes in PMBCL (≥ 5%) based on preliminary results of 22 whole-exome sequenced cases (n=46)

(iii) frequently amplified genes in PMBCL (n=5)

(iv) recurrently mutated genes in DLBCL (n=9)

(v) genes relevant to above target genes (n=12)

Bait design was performed targeting all coding regions. In addition, a set of baits targeting common SNPs was designed to allow for copy number calling. To improve the performance of the panel we added custom baits targeting regions that were poorly covered by conventional design and to regions of genes that were of high interest. The final panel consisted of a total of 20115 probes covering 0.91Mb of the genome. Information on the targeted genes in the panel and SNPs probes for copy number analysis is supplied in Suppl. Table S2.

By adding a significant number of whole-exome sequenced cases (n=57) until completion, a total of 16 driver genes that were identified in this study were not included in the final target panel.

Target sequencing

Library preparation for targeted sequencing was performed with the SureSelect$^{XT\ HS}$ custom capture bait kit (Agilent Technologies, Santa Clara, California, USA).

To ensure high quality, only samples that had a coverage of 100x in ≥ 80% of the exonic regions were included.

Variant calling was performed using Genomon2 pipelines (https://genomon.readthedocs.io/ja/latest/), as previously reported[6,7]. Briefly, sequencing reads were aligned to GRCh37 using BWA-mem version 0.5.8 with default parameter settings[8]. Biobambam2 was used for removal of PCR duplicates. Low quality and unreliable sequencing sequencing with a mapping quality of < 20 and bases with base quality scores < 15 were removed. Potential candidates were further filtered by following criteria:

for FFPE samples:

(i) sequencing depth ≥ 20

(ii) variant allele frequency (VAF) ≥0.05

(iii) *P*-value < $10^{-1.5}$ (Fisher realignment)

(iv) supported by reads mapped to both strands

for FF samples:

(i) sequencing depth ≥ 15

(ii) variant allele frequency (VAF) ≥0.05

(iii) P-value < $10^{-1.0}$ (Fisher realignment)

(iv) supported by reads mapped to both strands

The following adaptations were made as no germline controls were included:

(v) 10%_posterior_quantile >0.1

(vi) 10%_posterior_quantile(realignment) >0.1

(vii) VAF for synonymous and nonsynonymous SNVs <0.45, >0.55 and >0.95 for regions that were not affected by a SCNA.

To select variants that were observed at significantly higher VAFs than expected for errors, we used the following criteria: (viii) P-value $\leq 10^{-4}$ for FFPE, P-value $\leq 10^{-3}$ for FF samples and for which significance is evaluated by the EBcall algorithm[9] on the basis of an empirical distribution of VAFs as determined using sequencing data of non-paired lymphatic tissue FFPE samples of healthy controls (n=20). Furthermore, variants were further filtered when they were (iv) no registered in on of the following databases with a MAF >0.001: 1000 Genomes Project (May 2011 release), Exome Sequencing Project (ESP6500), Human Genome Variation Database (HGVD; April 2016 release), Human Genetic variation database (HGVD, http://www.genome.med.kyoto-u.ac.jp/SnpDB/), Exome Aggregation Consortium (ExAC), Genome Aggregation Database (gnomAD v2.1.1) and Genome Aggregation Database (gnomAD v2.1.1) exome.

In addition, mutations in putative hotspots were removed, if the ratio of excluded hotspot mutations / filtered hotspot mutations was >1. All previously excluded variants were rescued (e.g. acceptance of lower thresholds as described earlier), if

a) they were recurrently annotated (≥6 in one study or ≥3 in two independent studies) the Catalogue of Somatic Mutations in Cancer (COSMIC) databases for hematologic diseases v92

b) hotspots ≥ 3

c) detected in candidate driver genes

d) were putative loss-of-function mutations in known tumor suppressor genes.

In total, over 3000 filtered mutations were additionally inspected for artifacts and mapping errors through visual inspection with the Integrative Genomics Viewer (IGV)[10].

Two out of 157 samples did not have any mutations in the 106 genes analyzed via targeted resequencing and did not show any copy number alterations.

We compared tumor-only targeted sequencing data and paired tumor-germline whole-exome sequencing data from each individual case in 57 samples that were sequenced with both techniques for a corresponding study[11].

Notably, both WES, and targeted sequencing were carried out individually, both technically (e.g. library preparation) and bioinformatically for analysis and filtering or FFPE derived artifacts and sequencing errors as well as the principle bioinformatics pipeline, especially with focus on detecting

FFPE-derived sequencing errors by using FFPE derived lymphatic tissue as controls (so-called EB-call of the pipeline).

In detail, we used the raw sequencing reads of concurrently tumor and germline exome sequenced samples, to validate the filtered targeted-sequencing data, according to the following rules:
Mutations deemed true positives that were detected in the targeted sequencing and that had more than 10 reads in both tumor and normal samples were classified by the following criteria:
(i) somatic: supported by >2 reads in the tumor sample & VAFtumor > 0.01 & VAFtumor/VAFnormal > 5
(ii) germline SNP: VAFnormal > 0.15
(iii) sequencing error: supported by >2 reads in the normal sample & VAFnormal < 0.15 & VAFtumor/VAFnormal < 5
(iv) validation error: coverage >100x & no variant reads in the tumor sample
(v) low coverage: sequencing coverage < 10x in the tumor or normal sample
(vi) ambiguous: all others

In total 1911/2012 (94.9%) mutations were classified as somatic, 4 as germline SNPs, 5 as sequencing errors and 3 as validation errors. From the remaining 26 low coverage variants, a minimum of 7 are thought to be somatic as the mutation has been found to be somatic in other samples. Likewise, 29 of the remaining 63 variantss that were classified as ambiguous, were thought to be somatic as determined in other samples. In total, 1947/2012 mutations (96.8%) were classified as validated.

RNA Profiling on Nanostring nCounter

Total RNA of 137 PMBCL patients was isolated in cooperation with the Central Biomaterialbank of the Charité (ZeBanC) which was responsible for storage and processing of the biomaterials. Briefly, 5 µm specimens of FFPE conserved tumors were stained with hematoxylin and eosin to determine tumor area and content. Additional 7 to 10 5 µm specimens with a tumor content >50% were isolated by use of the Maxwell RSC RNA FFPE Kit on the Maxwell RSC 16 platform (Promega, Madison, WI, USA). RNA was eluated with 30 µl NF-water. RNA quality, yield and fragment distribution was determined on TapeStation 4200 (RNA Screen Tape; Agilent, Santa Clara, USA), Nanodrop ND-2000c (Peqlab/VWR, Radnor, PA, USA) and Qubit 4 Fluorometer (Thermo Fisher, Waltham, MA, USA). Concentration of RNA fragments with a size of 50 to 300 nt was normalized to 20 ng/µl and 5 µl were further processed according to the nCounter XT Assay user manual (MAN-10023-11, July 2016). The predesigned human nCounter PanCancer Pathways panel probe set (XT-CSO-PATH1, NanoString Technologies, Seattle, WA, USA), including 770 genes from 13 canonical pathways and selected reference genes, was expanded by inclusion of 30 additional probes, covering candidate gene transcripts and B cell-associated pathways (Suppl. Table S3). Hybridization of probes were carried out on a PCR system GeneAmp 9700 thermocycler (Applied Biosystems, Foster City, CA, USA) and cartridges were prepped on a GEN2 Prep Station and and scanned on a Digital Analyzer platform (NanoString

Technologies, Seattle, WA, USA) according to the manufacturer's instructions. Raw data was further processed using nSolver v4.0.70 (based on R-3.3.2) software as follows:

After importing raw data and annotation files, preliminary default quality control (QC) was performed during data import. Specifically, samples were excluded, which met one of the following criteria: countable fields of view (FOVs) in the Digital Analyzer were <75%, the binding density was outside the confidence range of 0.1 to 2.25 spots per square micron, the positive control linearity was below a R^2 of 0.95, or the standard deviation of the 0.5 fM positive control was less than two standard deviations higher than the mean of the negative control probes (refer to nSolver User Manual MAN-C0019-08 for further details). A second QC was performed during experimental set up. Here, all six internal positive controls were used to define a positive control geometric mean of all samples and exclude samples outside the range of a normalization factor 0.3 to 3. Threshold count value was set to 20. In this step, no samples were excluded. A third QC was based on housekeeper normalization. Specifically, mRNA content of 40 predefined housekeeping genes (refer to list of panel genes at nanostring.com) were used to calculate a geometric mean. Normalization factor was set to a confidence range of 0.1 to 10, and samples with a very low mRNA content, which did not meet these criteria were excluded from the analysis. Finally, n=120 samples were included into further analysis. An advanced analysis was conducted on non-normalized raw data by use of the nSolver Advanced Analysis (version 2.0.115) tool with no low count data omission and automatic housekeeper selection for normalization according to the built-in geNorm algorithm (refer to MAN-10030-03) [39]. Optimized differential expression (DE) analysis with Batch and Cartridge ID as well as B*CL7A, ARID1A, ARID5B, EZH2,* and *ZFHX4* set as possible confounders. Benjamini-Hochberg method was applied for multiple testing correction[12].

Transfection of human cell lines

Cells were cultured in antibiotic-free media for >24 h, harvested and counted. For ectopic expression of ZNF217 in ZNF217 knockout clones 1 x10^6 cells were electroporated with 10 µg of pcDNA3_n-Flag_ZNF217 vector in 100 µl transfection buffer R in the Neon Transfection System (Thermo Fisher Scientific, Waltham, MA, USA). Parameters used were voltage: 1300 V, pulse width: 20 ms, pulse number: 2. Cells were harvested 24 h after transfection for downstream experiments. For transfection with Cas9-RNPs to generate knockout cell lines, 1 x10^5 cells were mixed with Cas9-RNP and buffer T to obtain a 10 µl transfection volume. Electroporation settings were 1600 V, pulse width: 10 ms, pulse number: 3. After transfection, cells were cultured in 500 µl antibiotic-free media. Transfection efficacy was estimated by either parallel transfection of pcDNA3-EGFP into control cells and flow cytometry, differential expression of target protein according to western blot detection or via determination of variant allele frequencies (VAVs) of induced variants in the cell bulk.

Flow Cytometry

To determine cell viability, cell pellets were resolved in PBS containing 1% BSA and 5 µM diamidino-2-phenylindole (DAPI; BioLegend, San Diego, Ca, USA). Cells were analyzed by flow cytometry (BD FACSCanto II, BD Biosciences, Franklin Lakes, NJ, USA).

For apoptosis determination assay, $3x10^5$ cells were diluted in ice-cold 1x Annexin-V binding buffer (10x buffer: 0.1M Hepes (pH 7.4), 1.4 M NaCl, and 25 mM CaCl2) and stained with anti-Annexin-V-FITC (1:30; BD Pharmingen, BD Biosciences, Franklin Lakes, NJ, USA) and 5 µM DAPI. 1 volume of 1x Annexin-V binding buffer was added after 15 min of incubation and cells were analyzed on FACSCanto II.

For cell cycle and mitotic index flow cytometry $3x10^5$ cells were washed in ice-cold PBS, fixed with 70% ice-cold Ethanol and stored at 4°C for 30 min up to one week. For staining, supernatant was removed and cells were washed in ice-cold PBS containing 0.5% Triton-X and 1% BSA. Primary antibody (phospho-Histone H3 (Ser10) XP Rabbit mAb, clone D2C8, Cell Signaling, Danvers, MA, USA) was applied 1:1600 for 1 h at room temperature in PBS/Triton-X/BSA. Cells were washed with PBS/Triton-X/BSA and secondary antibody (Alexa Fluor 488 goat anti-rabbit IgG, RRID: AB_143165, Invitrogen, Carlsbad, CA, USA) was applied 1:500 in PBS/Triton-X/BSA for 1 h at room temperature. Cell were washed with PBS/Triton-X/BSA, supernatant was removed and resuspended in 0.5 ml PBS containing 20 µg/ml propidium iodide (Sigma-Aldrich) and 10 µg/ml RNase A (R4642, Sigma-Aldrich). After 20 min incubation on room temperature, cells were analyzed on FACSCanto II.

For determination of MHC class II presentation, $2x10^5$ cells were stained in PBS/1% BSA with the following antibodies (1:50): FITC Mouse Anti-Human HLA-DR, DP, DQ, Clone Tu39 (RUO) and FITC Mouse IgG2a, κ Isotype Control, Clone G155-178 (RUO) (both BD Biosciences, Franklin Lakes, NJ, USA) for 15 min., washed, counterstained with DAPI and analyzed on FACSCanto II. Flow cytometry data was analyzed with FlowJo v10.6.1 and Prism v9.1.0.

RNA Sequencing

For RNA-sequencing-based gene expression profiling, single cell clones with wild type and knock out ZNF217 genotype were cultured under standardized parameters (37°C, 5% CO2, identical LOTs of supplemental FBS and media, starting concentration: 1.8 $x10^5$ viable cells/ml) for three days. Cells were harvested and RNA and DNA was obtained by use of AllPrep DNA/RNA Mini Kit including DNAase treatment (Qiagen, Hilden Germany) according to the manufacturer's recommendation. Quantification and quality control was carried out on TapeStation 4200 (RNA Screen Tape; Agilent, Santa Clara, USA) and Qubit 4 Fluorometer (Thermo Fisher, Waltham, MA, USA) devices. For library preparation, 500 ng of total RNA was processed by use of the TruSeq RNA Library Preparation Kit v2 Set A (Illumina, San Diego, CA, USA) following to the TruSeq RNA Sample Preparation v2 Guide #15026495 F (Low Sample (LS) protocol). Briefly, mRNA was denatured, purified over bead separation and fragmented. First strand synthesis was carried out by reverse transcription using Superscript II reverse transcriptase (Invitrogen, Carlsbad, CA, USA). After second strand synthesis and clean up, end repair, A-tailing of 3' ends and adapter ligation (TrueSeq RNA adapter indices Set A) was performed according to the protocol. After cleanup, 1 µl of library cDNA mix was analyzed by real-time qPCR in a StepOnePlus Real-Time PCR device (Applied Biosystems, Foster City, CA, USA) by use of Luna Universal qPCR Master Mix (NEB, Ipswich, MA, USA). Subsequently, 10 cycles of library amplification was carried out according to the TrueSeq protocol. After cleanup, libraries were

quantitated and quality controlled on Tapestation 4200 (using the D1000 tapes, Agilent, Santa Clara, USA) and Qubit 4.

Sequencing was carried out after equimolar pooling on the NextSeq500 platform (Illumina, San Diego, CA, USA) by use of the High Output flowcell (Illumina, San Diego, CA, USA) on 76 bp paired-end mode. Demultiplexing was performed using bcl2fastq v2.20.0.

Differentially expressed genes were identified using the DESeq2 R package. Differentially expressed genes were defined by fold-change and q values (log2FC <-0.5 or >0.5; q<0.05). Gene set enrichment analysis (GSEA) was performed by use of GSEA 4.1.0 with a log2fold-change preranked list and using the CERNO algorithm implemented in the tmod package (v. 0.46.2)[13]. For GSEA, gene list was tested against each set collection (C3:TFT, C2:CP:Reactome, Hallmark, C5:GO, and a custom gene set collection associated with the perturbation of REST[4,5], HDAC3[14] and LSD1/KDM1A[15] (Suppl. Table S8) or MSigDB C2 or hypothesis-driven selected signatures derived from MSigDB C7 and literature (defined in Suppl. Table S8) separately. For CERNO gene set enrichment analysis, gene sets defined in the tmod package as well as a subset of gene sets defined in the MSigDB[16] and a number of predefined gene sets were used. The MSigDB gene sets were sourced from the msgidbr package, v. 7.4.1 and included REACTOME and KEGG pathways, HALLMARK gene sets, miRNA targets and GO categories from the biological pathway ontology. Custom genes sets were chosen hypothesis-driven and included gene sets derived from perturbation studies on chromatin modifying proteins. Normalized enrichment scores of GSEA and p-values were plotted with Prism 9.1.0.

Mass-Spectrometry LC-MS/MS

For global and phosphoproteomic profiling, cells were cultured under identical standard conditions for four days. After counting and re-adjustment of the cell density, another period of three days of identical culturing was carried out. Cells were counted and 2 $x10^7$ living cells were washed in ice-cold phosphate-buffered saline (PBS).

For relative quantification of the global proteome and phosphoproteome by LC/MS-MS, cell pellets were lysed in SDC lysis buffer (1% Sodium deoxycholate, 150mM NaCl, 50mM Tris-HCl pH 8, 1mM EDTA, 10mM DTT (dithiothreitol, Sigma), 40mM CAA (2-chloroacetamide, Sigma), Phosphatase inhibitor cocktail II and III (Sigma)). Samples were incubated at 95°C for 10 min, cooled down and treated with Benzonase® (Merck, 50 units) for 30 min at 37°C. Protein concentrations were determined using the Pierce BCA assay. Extracted proteins were digested with endopeptidase LysC (Wako) and sequence-grade trypsin (Promega) overnight at 37°C with an enzyme-to-protein ratio of 1:100. The resulting peptides were desalted on C18 SepPak columns (Waters, 100mg/1cc) and dried down using a SpeedVac Concentrator (Savant SC210A). Peptides were dissolved in 50 mM HEPES and 200ug of each sample were labeled with 11-plex tandem mass tag (Fisher Scientific) reagents as described (PMID: 30967486). The samples were randomly distributed an internal reference (composed of equal peptide amounts from all samples) was included for data analyses. TMT labeling was quenched with 1M Tris-HCl pH 8, and samples were combined, desalted on C18 SepPak columns (Waters, 200mg/1cc) and fractionated by high-pH reversed phase off-line chromatography

(1290 Infinity, Agilent) into 30 fractions. Of each fraction, 10 % of material was dried down directly and used for proteome analyses. The remaining 90 % was pooled into 15 fractions and used for phosphopeptide enrichment. Phosphopeptide enrichment was performed using Fe(III)-IMAC cartridges and the AssayMAP Bravo Platform (Agilent Technologies) according to manufacturer's instructions. Peptides were eluted with 1% ammonia, acidify with formic acid and dried down.
For LC-MS/MS measurements, peptides were reconstituted in 3% acetonitrile with 0.1% formic acid and separated on a reversed-phase column (20 cm fritless silica microcolumns with an inner diameter of 75 µm, packed with ReproSil-Pur C18-AQ 1.9 µm resin (Dr. Maisch GmbH)) using a 98 min gradient with a 250 nl/min flow rate of increasing Buffer B (90% ACN, 0.1% FA) concentration (from 2% to 60%) on a High Performance Liquid Chromatography (HPLC) system (Thermo Fisher Scientific) and analyzed on a Q-Exactive Plus instrument (Thermo Fisher Scientific). The mass spectrometer was operated in data-dependent acquisition mode using the following settings: full-scan automatic gain control (AGC) target 3 x 106 at 70K resolution; scan range 350–1500 m/z; Orbitrap full-scan maximum injection time 10 ms; MS2 scan AGC target 5 x 104 at 35K resolution; maximum injection time 50 ms (for non-enriched peptides) or 100 ms (for phosphopeptides); normalized collision energy 32; dynamic exclusion time 30 s; precursor charge state 2–7, 10 MS2 scans per full scan.
RAW data were analyzed using MaxQuant software package (v 1.6.3.4)[31]. The internal Andromeda search engine was used to search MS2 spectra against a decoy human UniProt database (release 2019-07) containing forward and reverse sequences, as well as ZNF217 mutant sequences. The search included variable modifications of methionine oxidation and N-terminal acetylation, deamidation (N and Q) and fixed modification of carbamidomethyl cysteine. Reporter ion MS2 for TMT11 was selected (internal and N-terminal) and TMT batch specific corrections factors were specified. For phosphopeptide enriched samples phosphorylation on S, T and Y was set as variable modification. Minimal peptide length was set to seven amino acids and a maximum of 3 missed cleavages was allowed. The FDR (false discovery rate) was set to 1% for peptide and protein identifications. Unique and razor peptides were included for quantification. The resulting text files were filtered to exclude reverse database hits, potential contaminants. For statistical data analysis the reporter ion intensity log2 ratio for each sample to the internal reference was calculated and z-score normalized (mean). Differences in protein abundance between ZNF217 variant cell lines were calculated using Welch t-test. Proteins passing the significance cut-off of < FDR5% were considered differentially expressed.

Cloning and Mutagenesis

ZNF217 cDNA sequence was generated from mRNA of K-562 cells by in-vitro transcription using the NEB HiScribe™ T7 High Yield RNA Synthesis Kit according to the manufacturer's recommendations and subsequent PCR amplification by a standard PCR protocol (30 cycles) with KAPA HiFi HotStart ReadyMix PCR Kit using the following primers:
ZNF217-cDNA-Fwd 5' CGA-TGATCA-CCGCCAGCAATCGAAAGTGACAGGAAACA 3'
NF217-cDNA-Rev 5' CTA-TGATCA-TCAAGTTTTTTGTCATTTGGTCGA 3'
ZNF217-3Fwd 5' TCTCAGAACGCATACAGGTGA 3'

ZNF217-3Rev 5' ACTTCAGCAGCAACATCGGT 3'

cDNA fragments were purified and combined by overlap extension PCR using the same PCR kit as in the previous step using just the ZNF217-cDNA-Fwd/Rev primer pair that also added flanking overhangs containing the restriction sites for BclI.

Full-length ZNF217 cDNA sequence was cloned into the multiple cloning site (MCS) of the pUC19 cloning vector (vector ID #50005, Addgene, Watertown, MA, USA). After purification the PCR product was digested with BclI (NEB, Ipswich, MA, USA). BamHI (NEB, Ipswich, MA, USA) was used to digest the target cloning vector pUC19 (vector ID #50005, Addgene, Watertown, MA, USA). Digestion products were verified by agarose gel electrophoresis, cleaned up by QIAquick Gel Extraction Kit (Qiagen, Hilden Germany) and ligated in a 1:3, 1:5 and 1:7 molar ratio (vector:insert) with Quick Ligation Kit (NEB, Ipswich, MA, USA) before transformation of DH5-alpha competent cells (Zymo Research, Irvine, CA, USA). Resulting clones were selected over 100 µg/ml ampicillin supplementation on agar plates. Correct sequence of the resulting pcDNA3-ZNF217 vector was verified after Miniprep (QIAPrep Spin, Qiagen, Hilden Germany) by Sanger sequencing (Eurofins Scientific, Luxemburg, Luxemburg).

For further subcloning, ZNF217 cDNA was PCR amplified by PCR standard protocol (30 cycles) with KAPA HiFi HotStart ReadyMix PCR Kit (KAPA Biosystems, Hoffmann-La Roche, Basel, Switzerland) using pUC19-ZNF217 as template. Primers generating flanking overhangs with restrictions sites for BclI and ApaI were: Fwd: 5' GCATGATCACCGCCATGCAATCGAAAGTGA 3' Rev.: 5' ATAGGGCCCTCAAGTTTTTTTGTCATTTGG 3'. PCR product was purified and digested with BclI and ApaI (NEB, Ipswich, MA, USA). Target vector pcDNA3-EGFP (vector ID #13031, Addgene, Watertown, MA, USA) was digested with BamHI and ApaI (NEB, Ipswich, MA, USA) to excise the eGFP Sequence and create compatible ends. Digestion products were verified by agarose gel electrophoresis, cleaned up by QIAquick Gel Extraction Kit (Qiagen, Hilden Germany) and ligated in a 1:3, 1:5 and 1:7 molar ratio (vector:insert) with Quick Ligation Kit (NEB, Ipswich, MA, USA) before transformation of DH5-alpha competent cells (Zymo Research, Irvine, CA, USA). Resulting clones were selected over 100 µg/ml ampicillin supplementation on agar plates. Correct sequence of the resulting pcDNA3-ZNF217 vector was verified after Miniprep (QIAPrep Spin, Qiagen, Hilden Germany) by sanger sequencing (Eurofins Scientific, Luxemburg, Luxemburg). The pcDNA3-ZNF217 vector was further modified to encode a Flag-tag fused ZNF217 protein. Therefore side-directed mutagenesis was carried out to generate a fusion protein with a Flag-tag on the n-terminus. To generate a n-terminal Flag and Linker, pcDNA3-ZNF217 was amplified with mutagenesis-specific primers (Fwd: 5'GACGACAAGGGAGGAGGAGGATCAATGCAATCGAAAGTGACAGGAAACATG3'; Rev: 5'ATCGTCTTTGTAGTCCATGGTGGCGGCGGTGATCCGAGCTCG3') by use of the Q5 Site-Directed Mutagenesis Kit (NEB, Ipswich, MA, USA) with minor modifications to introduce the following sequence upstream the ZNF217 ORF: 5' GCCACCATGGACTACAAAGACGATGACGACAAGGGAGGAGGAGGATCA 3'. This sequence encoded for a Kozak Sequence, the Start codon of the ORF, the DYKDDDDK Flag sequence, as well as the GGGGS-Linker Sequence. After transformation, selection and purification, vectors were verified by Sanger Sequencing. The H393* variant was induced into the pcDNA3-nFlag-ZNF217

vector using the same protocol with the following primers: Fwd: 5'GCTGGTCTTGtagTCCAGGGTCC3' and Rev: 5'TGGTGGTAGGTTCTGAAAGC3'.

Assay for Transposase-Accessible Chromatin with high- throughput sequencing (ATAC-Seq)

ATAC-sequencing was carried out by use of a previously published Omni-ATAC protocol[17] with minor modifications. Briefly, $1x10^8$ cells were separated from debris and dead cells over ficoll (Biochrom, Berlin, Germany) at room temperature and 400 RCF for 30 min. Overall viability was determined after DAPI staining by flow cytometry (BD FACSCanto II, BD Biosciences, Franklin Lakes, NJ, USA). If viability was below 90%, dead cells were depleted by use of a magnetic activated cell sorting (MACS)-based Dead Cell Removal Kit (#130-090-101, Miltenyi, Bergisch Gladbach, Germany) according to the manufacturer's instructions followed by a second flow cytometry run. $1x10^5$ cells with >90 % overall viability were used for lysis and transposase reaction following the Omni-ATAC protocol. PCR was performed on a Genetouch Thermal Cycler TC-E-96GA (BIOER Technology, Hangzhou, China) with 5 cycles of pre-amplification using a common Ad1 primer and specific Ad2 primers for single-indexing based on Illumina Nextera i7 adapter barcodes (Suppl. Table S6). Number of additional cycles was determined by real-time qPCR in a StepOnePlus Real-Time PCR device (Applied Biosystems, Foster City, CA, USA) with 5 µl of the PCR product according to an ATAC-seq library amplification procedure previously described[18]. Three further cycles of PCR were performed with the remaining PCR product without addition of further components. The final PCR product was cleaned up with 1.8x volume of AMPure XP magnetic beads (Beckman Coulter, Brea, CA, USA). Library fragment size distribution and quantification was carried out by use of TapeStation 4200 (Agilent, Santa Clara, USA) and Qubit 4 Fluorometer (Thermo Fisher, Waltham, MA, USA) devices. Libraries were equimolar pooled and sequenced on the Illumina NextSeq500 High Output NGS platform (Illumina, San Diego, CA, USA) in paired-end 2 x 76 mode. Demultiplexing was performed using bcl2fastq v2.20.0.

Sequencing reads were quality checked and adapter trimmed using FastQC. Reads were aligned to the hg19 human reference genome using Bowtie2 (RRID:SCR_016368). SAM files were converted into BAM format, sorted, de-duplicated and indexed with Picard (RRID:SCR_006525) and Samtools (RRID:SCR_002105). Reads mapping to repetitive regions or to the mitochondrial genome were discarded. ATAC-seq peaks were called using Macs2 (RRID:SCR_013291), accepting lambda but not model building. Differential peak enrichment was calculated with the DiffBind (RRID:SCR_012918) package in R.

For the data integration with RNASeq, differential ATAC peaks were annotated to their closest promoters and intersected with annotated differential RNASeq signals using bedtools intersect. Only regions with FDRq <0.05 for both ATAC and RNASeq signal differences and at least a log2 fold change of 0.5 for RNASeq were validated for later experiments. Promoter regions were defined as +/-2500bp of TSS. Motif discovery and analysis were performed with HOMER.

Co-immunoprecipitation and ZNF217 interactome analyses by mass spectrometry

Cells were harvested and lysed at 4°C in 1x cell lysis buffer (0.025M Tris, 0.15M NaCl, 0.001M EDTA, 1% NP-40, 5% glycerol; pH 7.4; Pierce™ Co-Immunoprecipitation Kit, Thermo Fisher Scientific, Waltham, MA, USA) supplemented with cOmplete™, Mini, EDTA-free Protease Inhibitor Cocktail (Roche, Basel, Switzerland) according to the manufacturer's protocol. To exclude indirect interaction via DNA binding, a control sample was benzonase-treated before further processing (Sigma-Aldrich, St. Louis, MO, USA). Lysates were incubated under continuous rotation with prewashed Anti-FLAG® M2 Magnetic Beads (Sigma-Aldrich) at 4°C overnight. To immobilize protein-bound beads, tubes were placed into a magnetic separation rack. Beads were washed twice with freshly prepared 1 ml TBS + 0.1 % NP40 and twice with 1 ml TBS. For quality control, 20% of the beads were used. Protein-antibody complexes were eluted by heat denaturation in 1x Laemmli sample buffer (Biorad, Hercules, CA, USA) including 10% beta-mercaptoethanol and analyzed by SDS-PAGE and western blot.

Beads from pulldown experiments were resuspended in 20 µl urea buffer (6 M urea, 2 M thiourea, 10 mM HEPES, pH 8.0), reduced for 30 minutes in 12 mM dithiothreitol solution, followed by alkylation by 40 mM chloroacetamide for 20 minutes at room temperature. Samples were first digested using 1 µg endopeptidase LysC (Wako, Osaka, Japan) for 3 hours, followed by dilution with 60 µl 50 mM ammonium bicarbonate (pH 8.5) and digestion with 1 µg trypsin (Promega) overnight at room temperature. The digestion was stopped by adding trifluoroacetic acid (1% final concentration). The peptides were extracted and desalted using StageTip protocol[19].

Peptides were eluted from stage tips using 80% Acetonitrile/ 0.1% formic acid, dried and resolved in 3% acetonitrile/0.1% formic acid. Peptide separation was performed on a reversed-phase column (20 cm fritless silica microcolumns, inner diameter of 75 µm, packed with ReproSil-Pur C18-AQ 3 µm resin (Dr. Maisch GmbH) coupled to an High Performance Liquid Chromatography (HPLC) system (Thermo Fisher Scientific), using a 90 minute gradient with a flow rate of 250 nl/min. Sample were ionized using an electrospray ionization (ESI) source (Thermo Fisher Scientific) and analyzed on an Q Exactive Plus instrument (Thermo Fisher Scientific), run in data dependent mode with a full scan in the Orbitrap (70K resolution; 3×10^6 ion count target; maximum injection time 50 ms, mass range 300-2000 m/z) followed by top 10 MS2 scans using higher-energy collision dissociation (17.5 K resolution, 5×10^4 ion count target; 1.6 m/z isolation window; maximum injection time: 250 ms). Only precursors with charge state 2-6 were sampled for MS2. Dynamic exclusion was set to 30 sec.

Data were analyzed using MaxQuant software package (version 1.6.3.4)[20]. The internal Andromeda search engine was used to search MS2 spectra against a decoy human UniProt database (HUMAN.2016-08) containing forward and reverse sequences. The search included variable modifications of methionine oxidation, N-terminal acetylation and deamidation (N and Q), as well as fixed modification of carbamidomethyl cysteine. Minimal peptide length was set to seven amino acids and a maximum of 3 missed cleavages was allowed. The FDR (false discovery rate) was set to 1% for peptide and protein identifications. Unique and razor peptides were considered for quantification. "Match between runs" option and LFQ normalization were activated. The resulting text files were filtered to exclude reverse database hits, potential contaminants, and proteins only identified by site. Statistical data analysis was performed using Perseus software (version 1.6.2.1). Technical and

biological replicates for each condition were defined as groups and intensity values were filtered for “minimum value of 3” in at least one group. After log2 transformation, missing values were imputed with random noise simulating the detection limit of the mass spectrometer. Differences in protein abundance between *ZNF217* variants and vector control samples were calculated using two-sample Student's t-test. Proteins passing the significance cut-off (FDR 20% or log2 fold change >2, p-value <0.05) were considered ZNF217 binders. Differential binders between the ZNF217 variants were defined two-sample Student's t-test and permutation-based FDR cut off of 30% (q-value <0.3).

Chromatin-immunoprecipitation followed by quantitative PCR (CHIP-qPCR)

Cells were harvested, counted to obtain 1x106 cells per condition, and DNA-crosslinked following the instructions of the iDeal ChIP-seq kit for Histones (Diagenode, Liège, Belgium) for 6 min. Cell lysis and chromatin shearing followed the kit’s protocol with 25 final shearing cycles. As an input sample, 1 µl of sheared chromatin was put aside. Magnetic immunoprecipitation was performed overnight at 4°C with protein A coated beads and antibodies against H3K27Ac (Abcam, Cambridge, UK), or H3K27me3, H3K4me2, H3K4me3, and controls: H3K4me3, Rabbit IgG (all: Diagenode, Liège, Belgium). Beads were immobilized in a magnetic rack, washed, de-crosslinked according to the manufacturer’s protocol and DNA was purified using AMPure XP magnetic beads (Beckman Coulter, Brea, CA, USA). Primers for genomic areas identified by ATAC Sequencing were generated with Primer3Plus (https://www.bioinformatics.nl/cgi-bin/primer3plus/primer3plus.cgi; last access: 15-02-2021; Suppl. Table S7) and validated against different concentrations of diluted CHIP-samples. Positive control for H3K27Ac, H3K4me3 and H3K4me2 was *GAPDH*, and negative control was *HOXA9* or *MYT1*. Positive control for H3K27me3 was *MYT1* and negative control was *GAPDH*. Quantitative PCR was carried out for one randomly assigned WT/KO pair on a StepOne Plus Real-Time PCR device (Applied Biosystems, Foster City, CA, USA) by use of Luna Universal qPCR Master Mix (NEB, Ipswich, MA, USA) and analyzed with the StepOneTM software v2.3. CHIP-qPCR data was quantified relative to input by using the percent input method.

Data analysis and statistics

In vitro data was analyzed using Prism 9 (v9.1.0; GraphPad Software, San Diego, USA). In general, parametric tests were chosen for datasets with assumption of normality. Normality was assumed after Shapiro-Wilk and Kolmogorov-Smirnov test. All differences were considered to be significant with alpha=0.05. For two-group comparisons, Students t-test was used for two groups of normally distributed data, respectively. Mann-Whitney test was applied for data with rejected assumption of normality. For multiple-group data, one- or two-way ANOVA was applied for normally distributed data or a mixed-effect model (REML) with Geisser-Greenhouse correction was used for normally distributed data with missing values. Friedmann test was used for non-normally distributed data. Multiple testing correction was dependent from the tested hypothesis: Dunnett’s test was applied for comparisons with one common control group. For multiple combined comparisons between groups, Tukey’s test was used. The mixed-effect-model was corrected with Sidak’s multiple comparisons test. Growth curves were fitted with an exponential (Malthusian) growth model using least squares

regression without further weighting. Extra sum-of-squares F test was used to compare non-linear regression data of cell proliferation.

Supplementary References

1 Basso K, Klein U, Niu H, Stolovitzky GA, Tu Y, Califano A *et al.* Tracking CD40 signaling during germinal center development. *Blood* 2004; **104**: 4088–4096.

2 Loh JW, Guccione C, Di Clemente F, Riedlinger G, Ganesan S, Khiabanian H. All-FIT: allele-frequency-based imputation of tumor purity from high-depth sequencing data. *Bioinformatics* 2020; **36**: 2173–2180.

3 Szklarczyk D, Kirsch R, Koutrouli M, Nastou K, Mehryary F, Hachilif R *et al.* The STRING database in 2023: protein-protein association networks and functional enrichment analyses for any sequenced genome of interest. *Nucleic Acids Res* 2023; **51**: D638–D646.

4 Wagoner MP, Gunsalus KTW, Schoenike B, Richardson AL, Friedl A, Roopra A. The transcription factor REST is lost in aggressive breast cancer. *PLoS Genet* 2010; **6**: e1000979.

5 Liang J, Tong P, Zhao W, Li Y, Zhang L, Xia Y *et al.* The REST gene signature predicts drug sensitivity in neuroblastoma cell lines and is significantly associated with neuroblastoma tumor stage. *Int J Mol Sci* 2014; **15**: 11220–11233.

6 Kakiuchi N, Yoshida K, Uchino M, Kihara T, Akaki K, Inoue Y *et al.* Frequent mutations that converge on the NFKBIZ pathway in ulcerative colitis. *Nature* 2020; **577**: 260–265.

7 Yokoyama A, Kakiuchi N, Yoshizato T, Nannya Y, Suzuki H, Takeuchi Y *et al.* Age-related remodelling of oesophageal epithelia by mutated cancer drivers. *Nature* 2019; **565**: 312–317.

8 Li H, Durbin R. Fast and accurate short read alignment with Burrows-Wheeler transform. *Bioinformatics* 2009; **25**: 1754–1760.

9 Shiraishi Y, Sato Y, Chiba K, Okuno Y, Nagata Y, Yoshida K *et al.* An empirical Bayesian framework for somatic mutation detection from cancer genome sequencing data. *Nucleic Acids Res* 2013; **41**: e89.

10 Barnell EK, Ronning P, Campbell KM, Krysiak K, Ainscough BJ, Ramirez C *et al.* Standard operating procedure for somatic variant refinement of tumor sequencing data. doi:10.1101/266262.

11 Noerenberg D, Briest F, Hennch C, Yoshida K, Nimo J, Hablesreiter R *et al.* S101: GENETIC AND EPIGENETIC FACTORS DRIVING PRIMARY MEDIASTINAL B-CELL LYMPHOMA PATHOGENESIS AND OUTCOME. *HemaSphere* 2022; **6**: 2.

12 Benjamini Y, Yekutieli D. The control of the false discovery rate in multiple testing under dependency. *Ann Stat* 2001; **29**: 1165–1188, 24.

13 Zyla J, Marczyk M, Domaszewska T, Kaufmann SHE, Polanska J, Weiner J. Gene set enrichment for reproducible science: comparison of CERNO and eight other algorithms. *Bioinformatics* 2019; **35**: 5146–5154.

14 Senese S, Zaragoza K, Minardi S, Muradore I, Ronzoni S, Passafaro A *et al.* Role for histone deacetylase 1 in human tumor cell proliferation. *Mol Cell Biol* 2007; **27**: 4784–4795.

15 Wang J, Scully K, Zhu X, Cai L, Zhang J, Prefontaine GG *et al.* Opposing LSD1 complexes function in developmental gene activation and repression programmes. *Nature* 2007; **446**: 882–887.

16 Liberzon A, Subramanian A, Pinchback R, Thorvaldsdottir H, Tamayo P, Mesirov JP. Molecular signatures database (MSigDB) 3.0. Bioinformatics. 2011; **27**: 1739–1740.

17 Corces MR, Trevino AE, Hamilton EG, Greenside PG, Sinnott-Armstrong NA, Vesuna S *et al.* An improved ATAC-seq protocol reduces background and enables interrogation of frozen tissues. *Nat Methods* 2017; **14**: 959–962.

18 Buenrostro JD, Wu B, Chang HY, Greenleaf WJ. ATAC‐seq: A Method for Assaying Chromatin Accessibility Genome‐Wide. Current Protocols in Molecular Biology. 2015; **109**. doi:10.1002/0471142727.mb2129s109.

19 Rappsilber J, Ishihama Y, Mann M. Stop and go extraction tips for matrix-assisted laser desorption/ionization, nanoelectrospray, and LC/MS sample pretreatment in proteomics. *Anal Chem* 2003; **75**: 663–670.

20 Tyanova S, Temu T, Cox J. The MaxQuant computational platform for mass spectrometry-based shotgun proteomics. *Nat Protoc* 2016; **11**: 2301–2319.
